# ZapA uses a two‐pronged mechanism to facilitate Z ring formation in *Escherichia coli*

**DOI:** 10.1002/mlf2.70037

**Published:** 2025-12-21

**Authors:** Yuanyuan Cui, Han Gong, Di Yan, Hao Li, Wenjie Yang, Ying Li, Xiangdong Chen, Joe Lutkenhaus, Sheng‐You Huang, Xinxing Yang, Shishen Du

**Affiliations:** ^1^ State Key Laboratory of Metabolism and Regulation in Complex Organisms, College of Life Sciences Wuhan University Wuhan China; ^2^ Hubei Key Laboratory of Cell Homeostasis, College of Life Sciences Wuhan University Wuhan China; ^3^ Key Laboratory of Polar Environment Monitoring and Public Governance (Wuhan University), Ministry of Education Wuhan China; ^4^ Division of Life Sciences and Medicine University of Science and Technology of China Hefei China; ^5^ School of Physics Huazhong University of Science and Technology Wuhan China; ^6^ State Key Laboratory of Virology and Biosafety, College of Life Sciences Wuhan University Wuhan China; ^7^ Department of Microbiology, Molecular Genetics and Immunology University of Kansas Medical Center Kansas City Kansas USA

**Keywords:** bacterial cell division, Z ring, FtsZ, ZapA, Z ring organization

## Abstract

The tubulin‐like protein FtsZ assembles into the Z ring that leads to the assembly and activation of the division machinery in most bacteria. ZapA, a widely conserved protein that interacts with FtsZ, plays a pivotal role in organizing FtsZ filaments into a coherent Z ring. Previous studies revealed that ZapA forms a dumbbell‐like tetramer that binds cooperatively to FtsZ filaments and aligns them in parallel, leading to the straightening and organization of FtsZ filament bundles. However, how ZapA interacts with FtsZ remains obscure. Here, we reveal that ZapA uses a two‐pronged mechanism to interact with FtsZ to facilitate Z ring formation in *Escherichia coli*. We find that mutations affecting surface‐exposed residues at the junction between adjacent FtsZ subunits in a filament as well as in an N‐terminal motif of FtsZ weaken its interaction with ZapA in vivo and in vitro, indicating that ZapA binds to these regions of FtsZ. Consistent with this, ZapA prefers FtsZ polymers over monomeric FtsZ molecules and site‐specific crosslinking confirmed that the dimer head domain of ZapA is in contact with the junction of FtsZ subunits. As a result, disruption of the putative interaction interfaces between FtsZ and ZapA abolishes the midcell localization of ZapA. Taken together, our results suggest that ZapA tetramers grab the N‐terminal tails of FtsZ and bind to the junctions between FtsZ subunits in the filament to straighten and crosslink FtsZ filaments into the Z ring.

## Impact statement

Bacterial cell division requires a large protein complex known as the divisome, which is organized by the Z ring consisting of polymers of the bacterial tubulin FtsZ. Assembly of a functional Z ring relies on FtsZ‐associated proteins (Zaps) that organize FtsZ filaments properly within the Z ring. However, how these proteins work remains incompletely understood. Here, we reveal the molecular mechanism by which ZapA, a highly conserved Zap protein, promotes Z ring formation in *Escherichia coli*. Our findings provide critical insights into Z ring organization, with broad implications for bacterial cell biology and antibacterial therapeutic development.

## INTRODUCTION

Cell division is one of the most fundamental processes of life and the unique features of this process in bacteria make it a promising target for the development of novel antibiotics. It starts when polymers of the bacterial tubulin FtsZ coalesce into the Z ring at the future division site, which not only functions as a scaffold for the assembly of the divisome complex but also as a guide for septal peptidoglycan (sPG) synthesis^1^, ^2^, ^3^, ^4^, ^5^. Time‐lapse imaging of FtsZ fluorescent protein fusions in model organisms such as *Escherichia coli* and *Bacillus subtilis* shows that FtsZ filaments first form a diffuse structure at midcell, and then coalesce into a condensed ring with a defined width of about 80–100 nm and a thickness of about 40–50 nm^6^, ^7^, ^8^. Interestingly, super‐resolution microscopic analysis of the Z ring revealed that it is a highly dynamic discontinuous ring‐like structure consisting of clusters of FtsZ filaments anchored to the membrane^9^, ^10^, ^11^. Moreover, FtsZ filaments undergo treadmilling, a directional motion in which subunits add at one end and are released from the opposite end, driven by GTP‐dependent polymerization and GTPase‐dependent depolymerization of FtsZ, respectively^2^, ^3^. Recent studies found that the treadmilling dynamics of FtsZ filaments is important for them to condense into a mature Z ring and distribute sPG synthetic complexes, consisting of FtsQLBWI, around the division site to construct a smooth septum^7^, ^8^, ^12^, ^13^. However, how FtsZ filaments are organized within the Z ring and how their dynamics are coupled with sPG synthesis remain to be elucidated.

In in vitro reconstitution systems, FtsZ filaments tethered to the membrane, either by its natural membrane anchors or by adding a membrane‐targeting sequence, can self‐organize into ring‐like structures on supported membranes or in liposomes^14^, ^15^, ^16^, ^17^. However, these reconstituted Z rings are not as condensed and organized as the Z rings in vivo, suggesting that additional factors are necessary for the formation of functional Z rings inside cells. Indeed, it is well demonstrated that many FtsZ binding proteins, which can crosslink FtsZ filaments into organized structures in vitro, play a critical role in Z ring maturation in vivo^7^, ^18^, ^19^. In *E. coli*, a group of proteins named FtsZ‐associated proteins (Zaps: ZapA, ZapB, ZapC, ZapD, and ZapE) has been identified. Each of these proteins, except for ZapB, which affects Z ring formation indirectly via ZapA, can crosslink FtsZ filaments into large bundles in vitro^18^, ^20^, ^21^, ^22^, ^23^, ^24^, ^25^, ^26^, ^27^. The absence of ZapA or ZapB causes abnormal septa and a slight delay in cell division, but the absence of multiple Zap proteins results in a deficiency in forming condensed Z rings, leading to a severe division defect^18^, ^19^. On the other hand, overexpression of any one of these Zap proteins prevents normal Z ring formation in *E. coli*, suggesting that excessive crosslinking of FtsZ filaments is detrimental for proper Z ring organization. In *B. subtilis*, the absence of some FtsZ binding proteins also results in severe defects in Z ring condensation, ultimately leading to divisome assembly and sPG synthesis defects^7^. The absence of Zaps in other bacterial species, such as *Caulobacter crescentus* and *Streptococcus pneumoniae*, has also been reported to cause aberrant Z ring formation^28^, ^29^, suggesting that use of FtsZ crosslinkers to facilitate Z ring organization is widespread in diverse bacterial species.

ZapA is a very widely conserved FtsZ‐associated protein within the bacterial kingdom. It was first discovered in *B. subtilis* as a suppressor of MinD overexpression, which prevents Z ring formation and causes lethality^20^. Although ZapA is not essential for division, it is synthetic lethal with EzrA or DivIVA in *B. subtilis*, which are positive regulators of Z ring formation, and synthetic sick with other *zap* genes in *E. coli* ^18^, ^19^, ^20^, ^30^, ^31^. Importantly, ZapA turns out to be a part of the Ter linkage that promotes and stabilizes the Z ring as well as modulates constriction dynamics in *E. coli* ^32^, ^33^, ^34^. The Ter signal is a multilayer protein network mediated by ZapA, ZapB, and MatP which is bound to the terminus region of the chromosome and organizes it into a macrodomain^32^, ^35^. ZapA and ZapB form a cloud‐like structure at midcell independent of FtsZ by linkage to MatP and promote rapid formation of the Z ring^36^. DapE, a critical enzyme for the synthesis of the peptidoglycan precursor diaminopimelic acid (DAP), strengthens the Ter signal in a ZapB‐dependent fashion^37^. In *C. crescentus*, the Ter signal consists of ZapA, ZauP, and ZapT; the latter two are functional homologs of ZapB and MatP, respectively^28^, ^38^, ^39^. In the absence of ZapA, ZapB, or MatP, FtsZ filaments tend to form loose spiral ring‐like structures instead of condensed Z rings, resulting in delayed division and twisted septa^19^, ^21^, suggesting an important role for ZapA (the Ter signal) in organizing the Z ring.

ZapA forms a dumbbell‐like tetramer with a dimer head on each end, which can bridge FtsZ filaments to form ladder‐like structures and bundles in vitro^40^, ^41^. Tetramerization is essential for ZapA function, as mutations disrupting tetramer formation inactivate ZapA^42^, ^43^. Several studies have found that ZapA interacts with FtsZ in a 1:1 ratio and mutations in the dimer head of ZapA reduce its interaction with FtsZ^40^, ^41^, ^44^, ^45^, ^46^, suggesting that the dimer head is the binding site for FtsZ. However, where and how a ZapA tetramer binds to FtsZ still remains a mystery. If each subunit of the ZapA tetramer binds to an FtsZ molecule, the two ZapA monomers in a dimer head need to bind distinct sites on two adjacent FtsZ molecules in a filament because of the twofold symmetry of the ZapA dimer. Also, given the symmetry of the bipolar ZapA tetramer, the orientations of the two FtsZ filaments have to be anti‐parallel^40^. However, this is in conflict with the directional motion of FtsZ filament patches in mature Z rings as observed by advanced light microscopy and simulation studies^2^, ^7^, ^47^. A recent in vitro reconstitution study of FtsZ filament networks with purified FtsZ, FtsA, and ZapA on supported membranes showed that ZapA tetramers align FtsZ filaments in a parallel manner, straighten FtsZ filament bundles, and increase the spatial order of the filament networks^46^. Moreover, ZapA binds to FtsZ filaments transiently and does not affect FtsZ filament length or treadmilling speed. These observations are in line with the treadmilling dynamics of FtsZ filament patches within the Z ring in vivo. However, it is difficult to postulate how ZapA can align FtsZ filaments in parallel without affecting treadmilling dynamics, given the lack of binding site information on FtsZ and the symmetry of the ZapA tetramer.

In this study, we investigated the interaction between FtsZ and ZapA and found that ZapA uses a two‐pronged mechanism to facilitate Z ring formation, in which ZapA tetramers bind to the N‐terminal motif of FtsZ and the junctions between FtsZ subunits to simultaneously straighten and crosslink parallel FtsZ filaments. This model reconciles the contradictory observations about ZapA and sheds new light on the organization of FtsZ filaments within the Z ring.

## RESULTS

### ZapA overexpression prevents Z ring condensation but not the treadmilling dynamics of individual filaments

**Figure 1:**
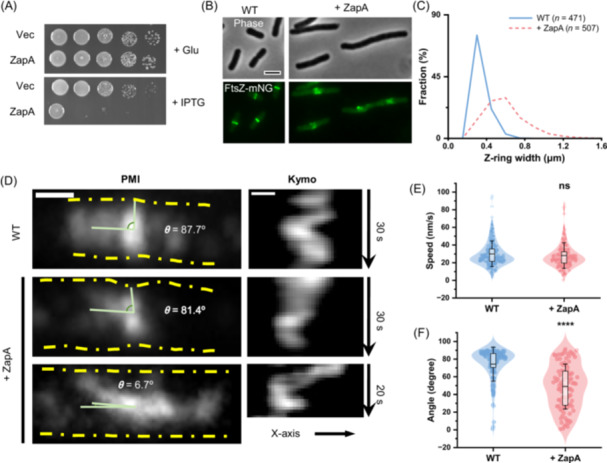
Overexpression of ZapA disrupts Z ring organization and inhibits cell division. (A) Overexpression of ZapA blocks colony formation. Plasmid pEXT22 or pSD319 (P_*tac*_::*zapA*) was transformed into strain W3110, and ZapA toxicity was assessed using a spot test. A 2 μl aliquot from each 10‐fold serial dilution was spotted onto LB plates with glucose (Glu) or IPTG and incubated at 37°C overnight before imaging. (B) Representative images of FtsZ‐mNG localization in the absence or presence of ZapA overexpression. Strain W3110 was transformed with a plasmid harboring *ftsZ‐mNG* under the control of an anhydrotetracycline‐inducible promoter and an empty vector or a plasmid carrying *zapA* under the control of an IPTG‐inducible promoter. FtsZ‐mNG was expressed at the basal level (without ATc), and ZapA was induced with 500 μM IPTG. Samples were taken and immobilized on an LB agarose pad for imaging. ATc, anhydrotetracycline. (C) Z ring width in the presence or absence of ZapA overexpression. Samples in (B) were analyzed as described in the Materials and Methods. Number of Z rings analyzed (WT: *n *= 471; + ZapA: *n* = 507) is shown. (D–F) Representative kymographs of FtsZ‐mNG (D) and computed FtsZ treadmilling velocity (E) and angles (F) in the absence or presence of ZapA overexpression. Number of filaments analyzed in (E) (WT: *n* = 203; + ZapA: *n* = 193) and number of angles analyzed in (F) (WT: *n *= 94; + ZapA: *n* = 97) are shown. The box plots (E, F) show the 25th and 75th percentiles as the box limits, with the mean at the center; whiskers extend from −SD to +SD of the mean. ns, not significant; ****p < 0.0001; two‐tailed Student's *t* test. Kymo, kymograph; PMI, projection of maximum intensity.

Overexpression of ZapA is known to result in the formation of aberrant FtsZ structures, which ultimately leads to a division block in *E. coli* ^48^, but how this occurs is not clear. To investigate this problem, we examined the localization dynamics of FtsZ upon ZapA overproduction. To do this, FtsZ‐mNeonGreen (FtsZ‐mNG) was ectopically expressed from a plasmid under the control of an anhydrotetracycline‐inducible promoter to monitor Z rings and ZapA was expressed from a second plasmid under the control of an IPTG‐inducible promoter (P_*tac*_::*zapA*) that provides sufficient ZapA to completely block colony formation in the presence of IPTG (Figure 1A). As expected, FtsZ‐mNG formed condensed rotating Z rings in cells in the absence of IPTG, but formed widened and distorted ring‐like structures at presumptive division sites within filamentous cells following induction of ZapA (Figure 1B,C, and Video S1). Interestingly, the treadmilling velocity of FtsZ filaments was not affected; however, instead of treadmilling perpendicular to the long axis of the cells, we found that the filaments treadmilled in various directions (Figure 1D–F). These observations indicate that too much ZapA alters the organization of FtsZ filaments and thus inhibits Z ring condensation, consistent with a recent report that ZapA changes the spatial order of the FtsZ filament network but does not affect FtsZ GTPase activity or filament length in vitro^46^.

Since Z ring condensation is critical for efficient recruitment of downstream division proteins to form the complete divisome and for sPG synthesis^7^, ^8^, ^19^, we checked how ZapA overexpression affected these processes. To do this, we examined the co‐localization of ZipA and FtsI, a proxy for the Z ring and a late division protein, respectively, and the co‐localization of ZipA and HCC‐amino‐D‐alanine hydrochloride (HADA), a fluorescent D‐amino acid for tracking nascent peptidoglycan (PG). We constructed a strain in which *zipA* was replaced with a functional *zipA‐mCherry* at its chromosomal locus and GFP‐FtsI was ectopically expressed from a plasmid under the control of an anhydrotetracycline‐inducible promoter. As shown in Figure S1A,B, the co‐localization of GFP‐FtsI and ZipA‐mCherry rings was evident in the absence of IPTG; however, the GFP‐FtsI signal was greatly reduced at the distorted ZipA‐mCherry rings upon the induction of ZapA. This was not due to cell filamentation because GFP‐FtsI still co‐localized with ZipA‐mCherry rings in filamentous cells caused by cephalexin, which blocks sPG synthesis by inhibiting FtsI (Figure S1A). Moreover, these aberrant structures appeared to be unable to support sPG synthesis efficiently, as indicated by the markedly reduced co‐localization of HADA with ZipA‐mCherry (Figure S1C,D). Thus, too much ZapA, just like the lack of ZapA, is detrimental for Z ring condensation, ultimately leading to defects in divisome assembly and sPG synthesis.

### Isolation of FtsZ mutants resistant to ZapA overexpression toxicity

To investigate how ZapA interacts with FtsZ, we isolated FtsZ mutants resistant to ZapA overexpression toxicity. This is possible since ZapA is necessary for efficient division but is not essential. We first constructed an FtsZ mutant library by replacing the wild‐type *ftsZ* in the plasmid pBANG112 with PCR random‐mutagenized *ftsZ* as previously described^49^, ^50^. The library was then introduced into the FtsZ depletion strain S17/pKD3C (W3110, *ftsZ* ^*0*^/pSC101^*Ts*^, *ftsZ*), which also harbored the plasmid pSD319 (pEXT22, P_*tac*_::*zapA*) for ZapA overproduction. Transformants were selected at 42°C in the presence of 30 μM IPTG, under conditions in which the pKD3C plasmid is lost and ZapA is overexpressed. Only transformants expressing FtsZ variants that are functional and resistant to overexpressed ZapA could survive (Figure S2A).

**Figure 2:**
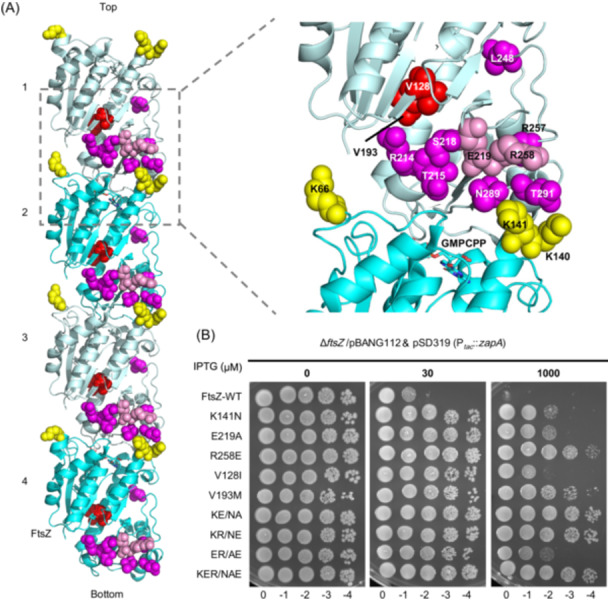
Mutations at the junction of FtsZ subunits in the filament confer resistance to ZapA overexpression. (A) Location of FtsZ mutations in the filament structure of *Klebsiella pneumoniae* FtsZ (PDB#: 8IBN). Residues on the surface of FtsZ are colored yellow (top face) or magenta (bottom face), whereas residues buried inside the FtsZ molecule are colored red. Note that mutations isolated by site‐directed mutagenesis are colored pink. GMPCPP is shown as a stick. Residue numbers are according to *Escherichia coli* FtsZ. (B) Spot test to assess the resistance of FtsZ mutants to ZapA overexpression. Plasmid expressing ZapA (pSD319) was transformed into strains expressing different FtsZ mutants from pBANG112 or its derivatives, and the transformants were subjected to a spot test on plates with or without IPTG. KE/NA, K141N and E219A; KR/NE, K141N and R258E; ER/AE, E219A and R258E; KER/NAE, K141N, R258E, K141N, E219A, and R258E.

Using the above approach, we isolated 15 transformants resistant to ZapA overexpression (Figure S2B). Sequencing *ftsZ* from the 15 pBANG112^M^ plasmids revealed that they all carried mutations in *ftsZ*, including 9 single mutations, 5 double mutations, and a triple mutation (Table S1). Subsequent analysis revealed that 13 single FtsZ substitutions provided resistance to ZapA overexpression (Figure S3A). The vast majority of these mutated residues are located on the surface of the polymerization domain, as shown on the monomeric structure of *E. coli* FtsZ (PDB#: 6UNX)^51^ (Figure S3B); however, two mutations, V128I and V193M, alter residues buried inside the FtsZ structure (Figure S3B). Moreover, the surface‐exposed mutations clustered at the top (the GTP binding pocket) or the bottom part of the FtsZ molecule (containing the T7 loop). A ZapA dimer head is unlikely to contact two sites so far apart in the molecule if it binds to an FtsZ monomer. Intriguingly, when these mutations were mapped onto the filament structure of FtsZ from *Klebsiella pneumoniae* ^52^, they clustered together at the junction of two adjacent subunits. Three mutations (V128I, V193M, and L248M) are a bit further away but likely affect the conformation of FtsZ (Figure 2A). These results indicate that ZapA may bind to a specific configuration of the junction between FtsZ subunits within a filament.

To test if additional mutations at the junction between FtsZ subunits conferred resistance to ZapA overexpression, we mutated additional residues in this region. Two more resistant mutations (E219A and R258E, both at the bottom part of the FtsZ molecule) were isolated (Figure S4A), suggesting that the junction between FtsZ subunits indeed contains residues important for interaction with ZapA. Overall, mutations affecting 14 residues were found to provide resistance to ZapA overexpression, with substitutions at 9 residues providing strong resistance, while substitutions at the other 5 residues provided modest resistance (Table S2). Since K141N, E219A, and R258E provide strong resistance to ZapA overexpression and represent mutations on the top and bottom parts of the FtsZ molecule that constitute the putative interaction interface, they were chosen for further study (Figure 2A). V128I, which likely represents a different class of resistant mutation, since it is buried inside the structure, was also included for the analysis. Reintroduction of the above single mutations or a combination of mutations (KE/NA: K141N and E219A; KR/NE: K141N and R258E; ER/AE: E219A and R258E; and KER/NAE: K141N, R258E K141N, E219A, and R258E) into pBANG112 revealed that they did not affect the ability of FtsZ to complement the depletion strain S17/pKD3C at 42°C and confirmed that they conferred strong resistance to ZapA (Figures 2B and S4B). Additionally, cells expressing these FtsZ mutants were still sensitive to the overexpression of ZapC (Figure S5), a different Zap protein that also interacts with FtsZ, indicating that they are specifically resistant to ZapA.

### Z rings formed by FtsZ mutants are resistant to the disruption by ZapA overexpression

**Figure 3:**
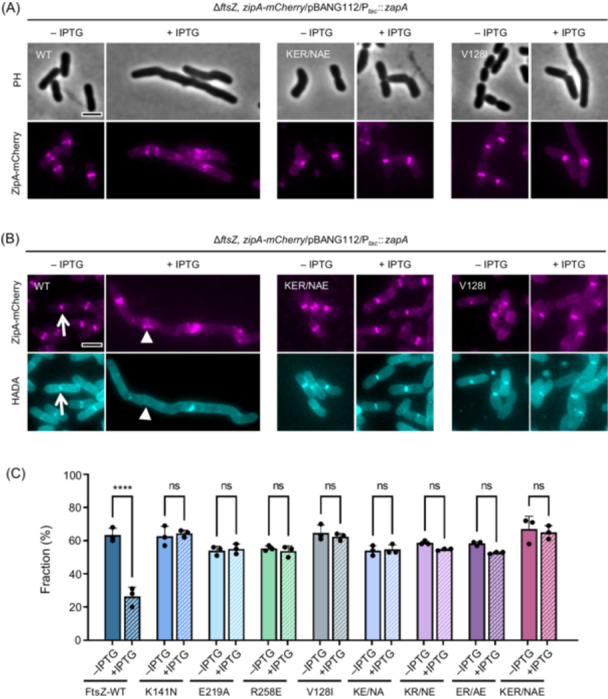
Z rings formed by FtsZ mutants are resistant to ZapA overexpression. (A) Representative images of Z rings (ZipA‐mCherry) in cells expressing wild‐type FtsZ or its variants in the absence or presence of ZapA overexpression. Cells expressing wild‐type FtsZ or its variants were grown in exponential phase, ZapA was induced with 500 μM IPTG, and ZipA‐mCherry was expressed from its native promoter. ZipA‐mCherry was imaged by fluorescence microscopy. PH, phase contrast. (B) Representative images of co‐localization of ZipA‐mCherry with HADA in cells expressing wild‐type FtsZ or its variants in the absence or presence of ZapA overexpression. Strains were grown as in (A); nascent PG was labeled with HADA as described in the Material and Methods section. White arrows and triangles indicate the normal and aberrant Z rings that overlap and do not overlap with a HADA signal, respectively. Note that sPG synthesis was blocked in cells expressing wild‐type FtsZ but not in cells expressing FtsZ mutants in the presence of overexpressed ZapA. HADA, HCC‐amino‐D‐alanine hydrochloride; PG, peptidoglycan. (C) Quantification of the co‐localization of ZipA‐mCherry and the HADA signal in (B). Data shown are the average of three experiments with more than 200 cells. Error bars indicate the SD. ns, not significant; ****p < 0.0001; two‐tailed Student's *t* test. Scale bars, 5 μm.

To explore the resistance phenotype of the FtsZ mutants to ZapA overexpression, we checked if the Z rings formed by these mutants could resist the disrupting effect of ZapA overexpression. To do this, *zipA‐mCherry* was introduced into the chromosome of strains expressing wild‐type FtsZ or the mutants using the λ‐Red recombineering system^53^. In the absence of IPTG to induce ZapA expression, ZipA‐mCherry localized at the division sites in condensed rings (Figure 3A). Upon overexpression of ZapA, cells expressing wild‐type FtsZ became filamentous, with ZipA‐mCherry forming disorganized spiral structures as expected. However, cells expressing FtsZ variants were much shorter and condensed ZipA‐mCherry rings were present in most cells, especially in the triple mutant (Figures 3A and S6A). These results demonstrate that resistance to ZapA overexpression is due to its reduced ability to disrupt Z rings formed by these FtsZ mutants. Moreover, quantification of the co‐localization of ZipA‐mCherry and the HADA signal revealed that ZapA overexpression decreased it from 63% to 26% in cells with wild‐type FtsZ, but it was not significantly reduced in cells expressing the FtsZ mutants (Figures 3B,C and S6B). Therefore, these FtsZ mutants form functional Z rings that are resistant to overexpressed ZapA, allowing normal divisome assembly and sPG synthesis.

### ZapA cannot crosslink FtsZ mutant filaments in vitro

**Figure 4:**
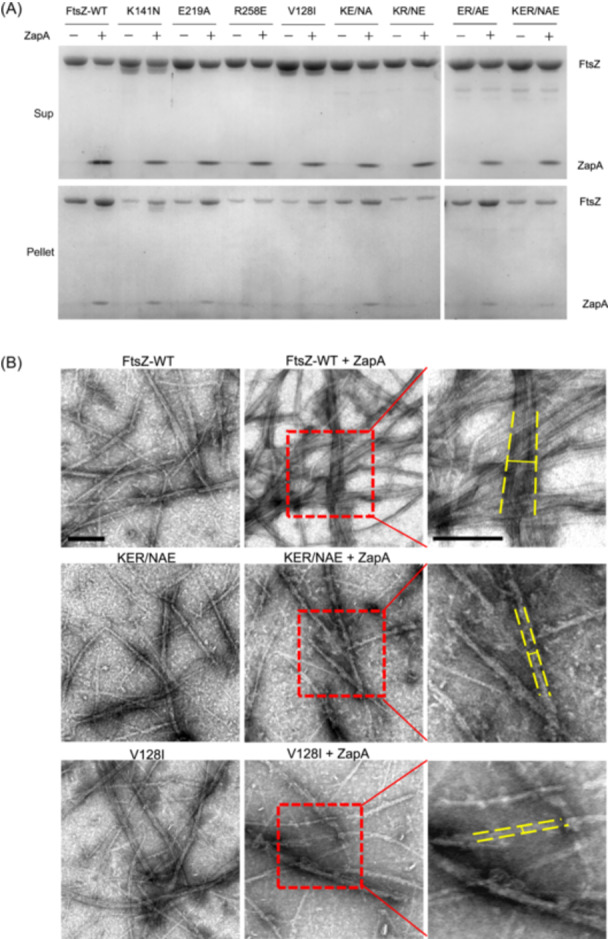
FtsZ mutants display resistance to ZapA in vitro. (A) Sedimentation assay to test the influence of FtsZ mutations on the crosslinking of FtsZ filaments by ZapA. FtsZ or its variants (5 μM) were mixed with or without equal molar ZapA in polymerization buffer (50 mM HEPES pH 6.8, 10 mM MgCl_2_, and 200 mM KCl) in the presence of GTP (2.5 mM) in a 50 μl reaction volume. The samples were incubated at room temperature for 5 min before being centrifuged. The pellets and supernatants were analyzed by SDS‐PAGE. (B) Negative stain electron microscopy analysis of the effect of the FtsZ mutations on the crosslinking of FtsZ filaments by ZapA. The reactions were performed as in (A), but the final concentration of proteins was lowered to 2.5 µM. GTP was added to a final concentration of 1 mM. Yellow lines show the width of the FtsZ filament bundles. Scale bar, 0.2 μm.

To confirm that the FtsZ mutants are resistant to ZapA overexpression, we purified them using the SUMO (small ubiquitin‐like modifier)‐tag protein purification system and examined the crosslinking activity of ZapA on their filaments. We first confirmed that the FtsZ mutants still polymerized by measuring their GTPase activity as previously described^50^, ^54^. The K141N and V128I mutations reduced the GTPase activity by about 50%, whereas the other two single mutations did not have an obvious effect (Figure S7A). For the double and triple mutants containing the K141N mutations, their GTPase activity was about 50%‐70% lower than that of wild‐type FtsZ. Nonetheless, the sedimentation assay showed that all the FtsZ mutants could be pelleted in the presence of GTP and Ca^2+^ (Figure S7B), indicating that they could still polymerize. The addition of ZapA led to a significant increase in the amount of wild‐type FtsZ and ZapA proteins in the pellet (done in the absence of Ca^2+^) (Figure 4A). However, ZapA had less of an effect on the amount of the FtsZ mutants in the pellet (Figures 4A and S8A). For some mutants, especially the triple mutant, ZapA had no effect, suggesting that ZapA could not crosslink these mutant FtsZ filaments effectively. In agreement with this, negative stain electron microscopy showed that FtsZ mutants formed single‐stranded filaments and small bundles similar to wild‐type FtsZ in the absence of ZapA, and ZapA promoted the formation of large bundles of wild‐type FtsZ filaments, but was unable to do so with the FtsZ mutants (Figures 4B and S8B). Thus, the FtsZ mutations provide resistance to the crosslinking activity of ZapA to various degrees, likely by reducing the binding affinity for ZapA.

### FtsZ mutations weaken the interaction between FtsZ and ZapA

**Figure 5:**
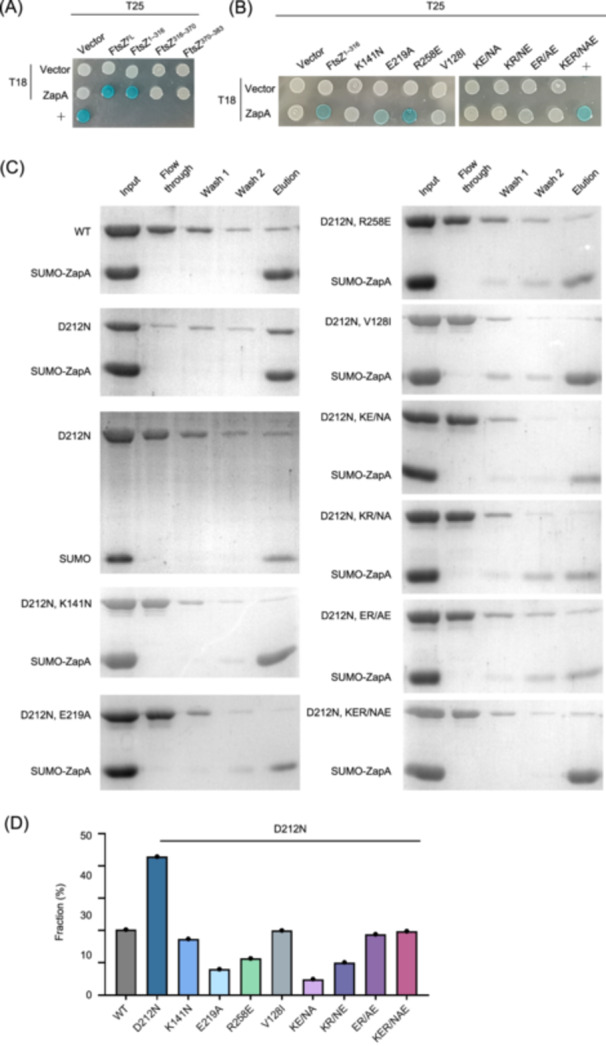
FtsZ mutations weaken the interaction between FtsZ and ZapA. (A) Bacterial two‐hybrid (BTH) assay to determine the domains important for FtsZ interaction with ZapA. Pairs of plasmids expressing the indicated fusions to the T18 and T25 domains of adenine cyclase were transformed into strain BTH101. A single transformant was resuspended in 1 ml of LB solution and spotted on LB plates containing IPTG and X‐gal. Plates were incubated at 30°C for 12 h before imaging. Blue indicates a positive interaction. +, positive control. A representative image from three independent experiments is shown. FtsZ^FL^, full length FtsZ. (B) BTH assay to access the impact of FtsZ mutations on the interaction between FtsZ and ZapA. Experiments were performed as in (A). (C) Pull‐down assay to determine the interaction between FtsZ mutants and ZapA. SUMO‐ZapA and FtsZ or its variants were incubated and treated according to the pull‐down assay described in the Materials and Methods section. All fractions were collected during the procedure and analyzed by SDS‐PAGE. Note that since the GTPase defective mutant FtsZ^D212N^ binds more readily to ZapA in vitro, the mutations were tested in the FtsZ^D212N^ background. (D) Quantification of the effect of mutations on the pull‐down efficiency of ZapA on FtsZ mutants in (C).

To examine the effect of the FtsZ mutations on the ZapA–FtsZ interaction in vivo, we used the bacterial two‐hybrid assay^55^. Since all the mutated residues are located in the globular domain of FtsZ, we constructed a truncated form of FtsZ (FtsZ^1–316^), which lacks the linker region (FtsZ^316–370^) and the C‐terminal tail (FtsZ^370–383^) of FtsZ, and tested its interaction with ZapA. As shown in Figure 5A, FtsZ^1–316^ interacted with ZapA as well as full‐length FtsZ, while FtsZ^316–370^ and FtsZ^370–383^ showed no interaction signal with ZapA, consistent with ZapA binding to the globular domain of FtsZ but not its linker or C‐terminal tail. Moreover, when the single mutations were introduced into FtsZ^1–316^, most of them greatly reduced or completely eliminated the interaction between FtsZ^1–316^ and ZapA (Figure 5B). All the double and triple mutations eliminated the interaction between FtsZ^1–316^ and ZapA, indicating that the mutated residues are critical for FtsZ interaction with ZapA in vivo.

To test the effect of the FtsZ mutations on the interaction between FtsZ and ZapA directly in vitro, we developed a pull‐down assay. The SUMO tag was removed from purified SUMO‐FtsZ but not from SUMO‐ZapA. Since our genetic results indicated that the putative binding site for ZapA only forms when FtsZ polymerizes into filaments or oligomerizes (Figure 2), GTP was added to the buffers throughout the experiments. When wild‐type FtsZ was incubated with SUMO‐ZapA, only a small amount of the protein co‐eluted with SUMO‐ZapA (Figure 5C,D), suggesting that wild‐type FtsZ interacts weakly with SUMO‐ZapA or FtsZ filaments turn over too fast to stably associate with it. If the latter was the case, then using a GTPase mutant (FtsZ^D212N^) that forms stable filaments should allow FtsZ filaments to associate more stably with ZapA. Indeed, when purified FtsZ^D212N^ was used for the pull‐down assay, it was enriched in the eluate with SUMO‐ZapA (Figure 5C,D). This was not due to nonspecific binding of stable FtsZ^D212N^ polymers to the Ni‐NTA column or the SUMO tag, as most of the protein was in the flow through when it was mixed with just the SUMO tag. We continued to test the effect of the mutations on the binding between FtsZ and ZapA after adding D212N to each of the mutants using the pull‐down assay. As shown in Figure 5C,D, FtsZ mutants were not enriched in the eluate with SUMO‐ZapA, indicating that the mutations weaken FtsZ's interaction with ZapA in vitro. Altogether, these results indicate that the mutated residues constitute the binding site for ZapA or are important for FtsZ filaments to adopt a correct conformation to interact with it.

### ZapA prefers polymerized FtsZ over monomeric FtsZ

Based on the above results, we hypothesized that an FtsZ mutant that could not polymerize would not interact with ZapA effectively. Previous studies reported that the introduction of mutations in the polymerization interface can lock FtsZ in the monomeric form, such as the L178E and L272E mutations^56^, ^57^. As expected, introduction of the L178E mutation into the T25–FtsZ^1–316^ fusions completely eliminated the interaction between FtsZ and ZapA in the bacterial two‐hybrid assay (Figure S9A). Moreover, almost the entire FtsZ^L178E^ protein was found in the unbound fraction when it was mixed with SUMO‐ZapA in the pull‐down assay (Figure S9B). These results strongly indicate that ZapA binds to FtsZ polymers more readily than FtsZ monomers.

### The ZapA dimer head is in contact with the junction between FtsZ subunits in the filament

**Figure 6:**
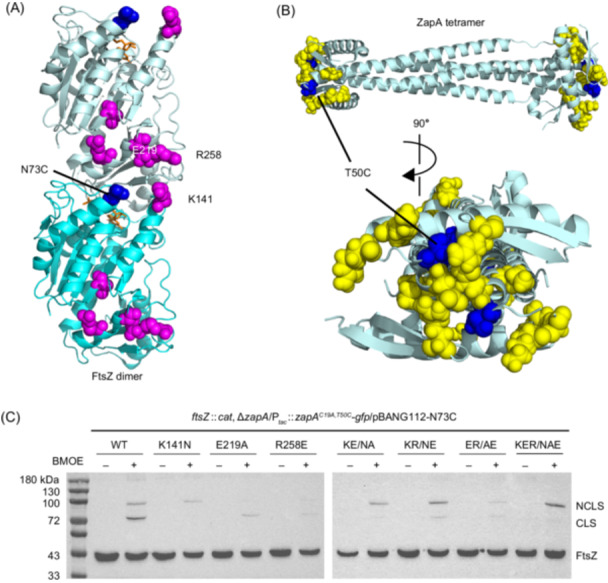
The ZapA dimer head contacts the junction between adjacent FtsZ subunits in the filament, as revealed by in vivo BMOE crosslinking assay. (A) Location of FtsZ mutations in an FtsZ dimer from the filament structure of *K. pneumoniae* FtsZ (PDB#: 8IBN). Residues affecting FtsZ interaction with ZapA are colored magenta and the cysteine mutation N73C is colored blue. GMPCPP is shown as a stick in brown. Residues' numbers are according to *E. coli* FtsZ. (B) Location of mutations in *E. coli* ZapA tetramer structure (PDB#: 4P1M). The cysteine mutation T50C is colored blue, whereas residues important for ZapA interaction with FtsZ are shown as spheres in yellow. (C) BMOE‐crosslinking assay to test the effect of FtsZ mutations on FtsZ's interaction with ZapA in vivo. Cells expressing FtsZ^N73C^ or its variants carrying the FtsZ mutations and ZapA^C19A,T50C^ were treated with BMOE or DMF for 15 min. Cells were then harvested by centrifugation and lysed in 1× SDS‐PAGE buffer, boiled for 10 min, and loaded onto SDS‐PAGE gel for western blot as described in the Materials and Methods section. BMOE, bis‐maleimidoethane; CLS, crosslinked species; DMF, dimethylformamide; NCLS, nonspecific crosslinked species.

The above results strongly indicate that ZapA binds to the exposed surface residues at the junction between adjacent FtsZ subunits in a filament. To confirm this, we tested if these regions of ZapA and FtsZ could be specifically crosslinked in vivo, and if so, what effect the resistant mutations had on the crosslinking. To do this, we introduced cysteine residues at the putative interaction interface (the junction between FtsZ subunits in a filament, and the dimer head of ZapA) and tested their effects on function to see if they would be appropriate for Cys crosslinking. Through screening a number of cysteine mutation pairs in FtsZ and ZapA, we found that the pair FtsZ^N73C^ and ZapA^T50C^ was suitable for the crosslinking experiments. The N73 residue of FtsZ is located close to the putative interaction interface with ZapA (Figure 6A). Moreover, complementation tests showed that the N73C mutation did not affect FtsZ's ability to support cell growth, even when it was combined with the mutations affecting FtsZ's interaction with ZapA (Figure S10A). Also, N73C did not provide resistance to ZapA overexpression (Figure S10B), suggesting that it does not affect interaction with ZapA. The T50 residue of ZapA was chosen for Cys substitution because it is located in the ZapA dimer head and adjacent to many residues important for ZapA's interaction with FtsZ^41^ (Figure 6B). The endogenous C19 residue of ZapA was also changed to alanine to avoid potential interference. A ZapA–GFP fusion containing these two mutations was still toxic, indicating that they did not affect the interaction with FtsZ (Figure S10C). If the introduced cysteine residues on FtsZ and ZapA were indeed located in the interaction interface, they may be crosslinked with the thiol‐specific compound bismaleimidoethane (BMOE) to generate a crosslinked FtsZ–ZapA–GFP species that is expected to have a molecular weight of 80 kDa (FtsZ: 40 kDa; ZapA–GFP: 40 kDa). Indeed, we observed such a migrating species by western blot analysis for FtsZ (CLS: crosslinked species) when these cysteine‐containing proteins were co‐expressed in the presence of BMOE (Figures 6C and S10C), suggesting that the cysteine pair was close to the intermolecular interaction interface. We also detected a band with a molecular weight about 100 kDa, which likely represents FtsZ crosslinked to an unknown interaction partner containing a cysteine or possibly through an amine group (BMOE can also react with molecules with an amine group inefficiently). Consistent with this, the nonspecific crosslinked FtsZ product was also detected when FtsZ^N73C^ was expressed alone (Figure S10C). Strikingly, introduction of the mutations resistant to ZapA overexpression into FtsZ^N73C^ greatly reduced the 80 kDa CLS (Figure 6C), suggesting that they weaken the interaction between FtsZ and ZapA. For an unknown reason, the band around 100 kDa was differentially affected by the mutations, suggesting that some of the mutations might also affect FtsZ's interaction with another unknown protein. Nonetheless, these results confirm that the dimer head of ZapA is in contact with the junction between FtsZ subunits in the filament and mutations resistant to ZapA overexpression disrupt this contact.

### ZapA also binds to the N‐terminal tail of FtsZ

**Figure 7:**
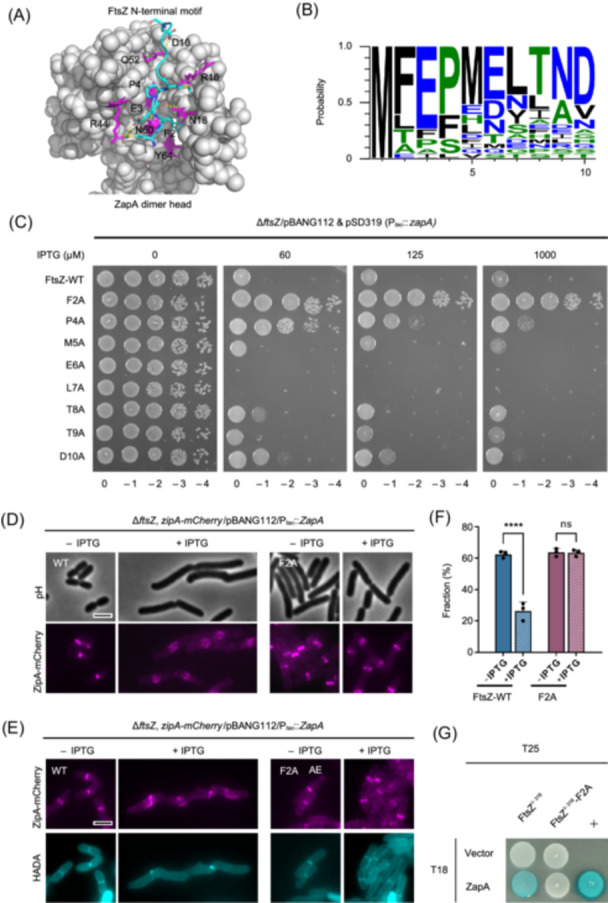
The N‐terminal motif of FtsZ is important for its interaction with ZapA in vivo. (A) AlphaFold 3 model of the FtsZ–ZapA complex indicates that the N‐terminal segment of FtsZ interacts with ZapA. The dimer head of ZapA is colored gray, while the N‐terminal motif of FtsZ is colored cyan. Residues important for interaction are indicated (FtsZ: F2, E3, P4, and D10; ZapA: R16, N18, R44, Q52, and N60). (B) Sequence logo of the N‐terminal motif of FtsZ across diverse bacterial species using Weblogo3. Alignment of the FtsZ N‐terminal sequences is shown in Figure S12A. (C) Spot test of the effect of mutations in the N‐terminal motif of FtsZ on the resistance to ZapA overexpression. A plasmid expressing ZapA (pSD319) was transformed into strains expressing different FtsZ mutants, and the transformants were subjected to a spot test on plates with or without IPTG. (D) Representative images of Z rings (ZipA‐mCherry) in cells expressing wild‐type FtsZ or FtsZ^F2A^ in the absence or presence of ZapA overexpression. (E) Representative images of co‐localization of ZipA‐mCherry with HADA in cells expressing wild‐type FtsZ or FtsZ^F2A^ in the absence or presence of ZapA overexpression. (F) Quantification of the co‐localization of ZipA‐mCherry and the HADA signal in (E). Data shown are the average of three experiments with more than 200 cells for each. Error bars indicate the SD of three experiments. ns, not significant; ****p < 0.0001; two‐tailed Student's *t* test. Scale bars, 5 μm. (G) BTH assay to test the impact of the F2A mutation of FtsZ on the interaction between FtsZ^1–316^ and ZapA. The test was carried out as shown in Figure 5A.

To further validate our results, we used AlphaFold 3 to predict the structure of the FtsZ–ZapA complex^58^. However, the prediction did not produce any high‐confidence structural models for the complex, regardless of the ratio between FtsZ and ZapA (2:2, 2:4, or 4:4). In fact, none of the models was able to predict the binding of ZapA to the junction between FtsZ subunits as deduced from the analysis above. Nonetheless, in all the models, the extreme N‐terminus of FtsZ (amino acids 1–10) inserts into a groove in the dimer head of ZapA (Figures 7A,B and S11), suggesting that this motif may be involved in binding to ZapA. Sequence analysis of FtsZ in a number of Gram^+^ and Gram^−^ bacteria revealed that this motif is quite conserved in bacteria encoding ZapA, featured by a large hydrophobic residue (F or L) and a glutamate residue at the second and third position, respectively (Figures 7B and S12A). To test if this motif is involved in interaction with ZapA, we mutated the first 10 amino acids of FtsZ by alanine scanning and tested whether the mutants provide resistance to ZapA overexpression toxicity. All mutants, except for the E3A substitution, were able to complement an FtsZ depletion strain (Figure S12B), suggesting that the E3 residue is critical for FtsZ function, whereas the other residues are not. Interestingly, the FtsZ^F2A^ and FtsZ^P4A^ mutants conferred strong and weak resistance to ZapA overexpression toxicity, respectively (Figure 7C). We therefore focused on the FtsZ^F2A^ mutant and found that Z rings (ZipA‐mCherry as a proxy) formed by this mutant were not disrupted by ZapA overexpression and sPG synthesis (labeled with HADA) was not significantly hindered (Figure 7D–F). Additionally, introduction of the F2A mutation into FtsZ^1‐316^ completely eliminated its interaction with ZapA in the BTH assay (Figure 7G). Thus, the N‐terminal motif of FtsZ is important for its interaction with ZapA in vivo.

**Figure 8:**
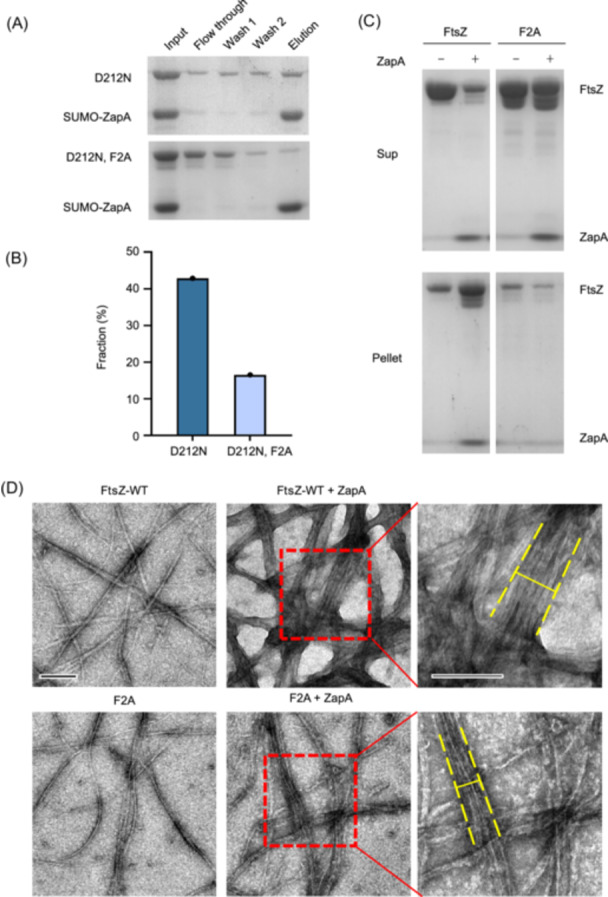
The F2 residue of FtsZ is important for its interaction with ZapA in vitro. (A) Pull‐down assay to assess the effect of the F2A mutation on FtsZ's interaction with ZapA. SUMO‐ZapA and FtsZ^D212N^ or FtsZ^D212N, F2A^ were incubated and treated according to the pull‐down assay described in the Materials and Methods section. All fractions were collected during the procedure and analyzed by SDS‐PAGE. (B) The effect of the F2A mutation on the pull‐down efficiency of FtsZ by ZapA quantified by measuring the fraction of FtsZ in the elution to the total amount of FtsZ in the input. (C) Sedimentation assay to test the impact of the F2A mutation on FtsZ's interaction with ZapA. The reactions were prepared as described in the Materials and Methods section with or without equal molar of ZapA. The samples were incubated at room temperature for 5 min before being centrifuged and the pellets and supernatants were analyzed by SDS‐PAGE. (D) Negative stain electron microscopy analysis of the effect of the F2A mutation on FtsZ interaction with ZapA. The reactions were performed as in (C), but the final concentration of proteins was lowered to 1 µM. GTP was added to a final concentration of 1 mM. The yellow line shows the width of FtsZ filament bundles. Scale bars, 0.2 μm.

To confirm that the N‐terminal motif of FtsZ was directly involved in the interaction between FtsZ and ZapA, we purified the FtsZ^F2A^ mutant and examined its interaction with ZapA. We confirmed that FtsZ^F2A^ polymerized by the sedimentation assay (Figure S12C), and then introduced the D212N mutation to carry out the pull‐down assay. As shown in Figure 8A,B, while FtsZ^D212N^ co‐eluted with SUMO‐ZapA, less FtsZ^D212N, F2A^ was co‐eluted, suggesting that the F2A mutation reduces FtsZ interaction with ZapA. Consistently, FtsZ^F2A^ sedimented less well in the presence of ZapA (Figures 8C and S12D). Also, while ZapA crosslinked wild‐type FtsZ filaments into large bundles, fewer bundles of FtsZ^F2A^ filaments were observed on the grids (Figure 8D). Moreover, the bundles formed by FtsZ^F2A^ and ZapA were much smaller and less organized in comparison to those formed by wild‐type FtsZ and ZapA. Together, these results demonstrate that F2 in the N‐terminal motif of FtsZ substantially contributes to the interaction between FtsZ and ZapA in vitro.

### Disruption of the interaction between ZapA and FtsZ prevents midcell localization of ZapA

**Figure 9:**
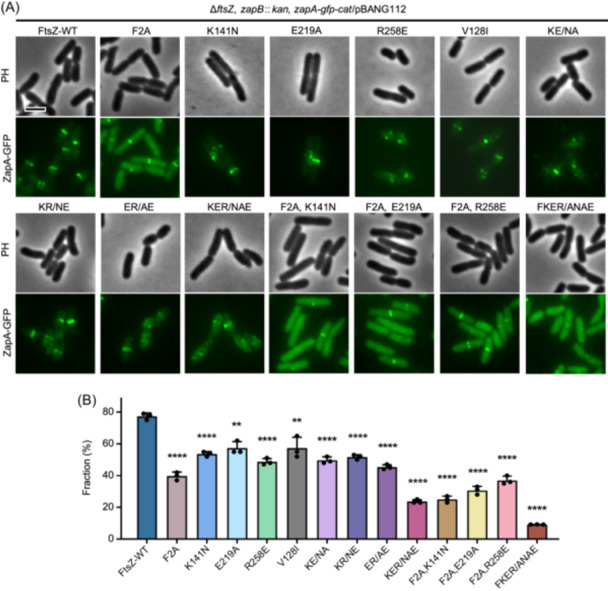
Localization of ZapA depends on its interaction with FtsZ. (A) Representative images of ZapA localization in cells expressing FtsZ mutants. Exponentially growing cultures of CYb1 (W3110, *ftsZ* ^*0*^ *zapA*‐*gfp*‐*cat zapB*::*kan*/pACYC, *ftsZ*) and derivatives carrying various *ftsZ* alleles were visualized by fluorescence microscopy to assess ZapA‐GFP localization at 30°C. Scale bar, 5 μm. (B) Quantification of the localization of ZapA‐GFP in cells expressing different FtsZ variants in (A). Data are presented as mean value ± SD. ****p < 0.0001; 0.0008 < **p < 0.0016; two‐tailed Student's *t* test. Number of cells analyzed (WT: *n* = 334; F2A: *n* = 836; K141N: *n* = 275; E219A: *n* = 352; R258E: *n* = 239; V128I: *n* = 242; KE/NA: *n* = 289; KR/KE: *n* = 347; KER/NAE: *n* = 876; F2A, K141N: *n* = 475; F2A, E219A: *n* = 918; F2A, R258E: *n* = 542; FKER/ANAE: *n* = 943) is shown.

Lastly, we tested if the presence of the FtsZ mutations prevented the localization of ZapA in vivo, since its localization depends upon its interaction with FtsZ. To do this, we introduced *zapA‐gfp* into the chromosome in the strains expressing wild‐type or FtsZ variants by the λ‐Red recombineering system^53^. We also deleted *zapB* in these strains because it interacts with ZapA to form an FtsZ‐independent cloud structure at midcell by a linkage to MatP^36^. As shown in Figure 9, ZapA‐GFP localized as a ring at the division site in close to 80% of cells expressing wild‐type FtsZ. However, it localized to midcell in only about 40% of the cells expressing the FtsZ^F2A^ mutant, accompanied by a substantial increase in cytoplasmic fluorescence. To our surprise, the single or double mutations at the junction between FtsZ subunits only modestly reduced midcell localization of ZapA‐GFP. Nonetheless, we did observe a strong reduction of ZapA‐GFP midcell localization in cells expressing the triple mutant (KER/NAE), where it formed discrete puncta or patches. The addition of any mutation at the junction to the F2A mutant further reduced the midcell localization of ZapA‐GFP and increased its cytoplasmic localization (Figure 9). More importantly, a combination of the triple mutations at the junction and the F2A mutation (FKER/NANE) almost completely eliminated the midcell localization of ZapA‐GFP. Thus, both the N‐terminal motif and the junction between FtsZ subunits in the filaments are important for ZapA binding to FtsZ in vivo, and disruption of both binding sites is necessary to eliminate their co‐localization.

## DISCUSSION

ZapA is a very widely conserved cell division protein that facilitates Z ring assembly in diverse bacteria, but its working mechanism remains unclear, in part because its binding site on FtsZ has not been determined. In this study, we found that mutations at the junction between adjacent FtsZ subunits within filaments and in an N‐terminal motif of FtsZ substantially reduced its interaction with ZapA, indicating that ZapA binds to both these regions of FtsZ. This dual binding mode enables ZapA to use the polymerization dynamics of FtsZ to crosslink FtsZ filaments into the Z ring. Moreover, we found that mutations internal to the FtsZ molecule can also significantly affect its interaction with ZapA, suggesting that ZapA recognizes a specific conformation of the filament. Taken together, our results suggest a model in which ZapA tetramers grab the N‐terminal tails of FtsZ and bind to the junctions between FtsZ subunits in filaments, such that they can straighten the longitudinal interaction of FtsZ like a staple and simultaneously crosslink FtsZ filaments in a parallel orientation.

The mechanism by which ZapA promotes Z ring assembly has been extensively investigated in vivo and in vitro. Early studies suggested that ZapA facilitates Z ring formation by crosslinking individual FtsZ filaments^20^, ^59^, but subsequent studies indicated that ZapA may be more important in aligning FtsZ clusters containing multiple filaments into condensed Z rings^21^. A recent in vitro reconstitution study of the effect of ZapA on the dynamic behavior of membrane‐bound FtsZ filaments provided important insight into its mechanism. It was shown that membrane‐bound FtsZ filament bundles self‐organize into swirling ring‐like structures, which reorganize into elongated bundles after the addition of ZapA^46^. Using high‐resolution fluorescence microscopy and quantitative image analysis, the authors showed that ZapA aligned FtsZ filaments in parallel, leading to the straightening and stabilization of the filament bundles, and thus the collapse of the swirling ring‐like structures^46^. However, ZapA only binds to FtsZ filaments transiently and has no effect on filament length or treadmilling velocity^46^. In line with these observations, we found that overexpression of ZapA in vivo did not affect the treadmilling speed of FtsZ filaments but altered their treadmilling orientation. Presumably, excess ZapA disrupts the proper curvature and orientation of FtsZ filaments so that they could not align properly into mature Z rings. Notably, these FtsZ filaments were still able to accumulate at potential division sites as disorganized structures in the filamentous cells, suggesting that they were still responsive to the regulation of the spatial regulators. These in vitro and in vivo observations are consistent with the idea that ZapA organizes the FtsZ filament network in the Z ring without affecting the dynamics of individual FtsZ filaments.

The crystal structures of ZapA showed that it forms an elongated anti‐parallel tetramer with two dimer heads connected by their C‐terminal coiled‐coils^40^, ^41^. The ZapA tetramer is able to crosslink FtsZ filaments into ladder‐like structures or large bundles, with each dimer head bound to a filament^20^, ^40^, ^45^. It may also stabilize the longitudinal interaction between FtsZ subunits within the filaments^20^, ^59^. However, how exactly ZapA interacted with FtsZ to achieve these functions was unknown. A previous study attempted to map the contact sites between ZapA and FtsZ in vitro by using chemical crosslinking coupled with mass spectrometry. They found that residues K51 and K66 of FtsZ were crosslinked to the K42 residue of ZapA^44^. Based on this crosslinking information and mutational analysis of ZapA, a model for the complex of the ZapA tetramer and FtsZ filaments was generated by docking, in which the ZapA dimer head binds to a region close to the polymerization interface of FtsZ^44^. However, this model is likely rudimentary because the crosslinking information is very limited.

In this study, we found that mutations altering residues in the junction between FtsZ subunits or internal to FtsZ confer resistance to ZapA overexpression, including K66, V128, K140, K141, V193, R214, T215, S218, E219, L248, R257, R258, N289, and T291. Notably, the K66 residue was identified in the previous crosslinking experiment^44^. Through in‐depth characterization of three representative residues in the junction region (K141, E219, and R258) by a combination of genetic, biochemical, and cellular approaches, we found that ZapA binds to the junction between FtsZ subunits in the filaments. This indicates that the interaction interface for ZapA is only available following FtsZ polymerization. Consistent with this, we showed that ZapA bound to stable FtsZ filaments (formed by the GTPase‐defective FtsZ^D212N^) better than dynamic wild‐type FtsZ filaments and did not bind to a monomeric mutant (FtsZ^L178E^). It is notable that a majority of the mutated residues affecting FtsZ's interaction with ZapA are charged residues, and many previously reported residues important for ZapA to interact with FtsZ are also charged. This indicates that the interaction between the dimer head domain of ZapA and the junction of FtsZ is largely via electrostatic interactions. It is also noticeable that the deduced binding site for ZapA covers a large surface‐exposed area at the junction between FtsZ subunits in a filament. This may explain why single mutations at this interface are not destructive enough to block the interaction completely, especially in vivo. In agreement with this, none of the reported single mutations in the dimer head of ZapA completely eliminated the interaction with FtsZ^41^. Also, the presence of the additional binding site for ZapA in the N‐terminus of FtsZ makes it difficult to eliminate ZapA localization to the Z ring in vivo.

A totally unexpected finding of this study is that ZapA also binds to an N‐terminal motif of FtsZ via a groove in the dimer head. This short motif, especially the conserved F at position 2 (*E. coli* numbering), is not important for FtsZ polymerization and is not present in structures of FtsZ. Also, this motif has not been implicated in interaction with any known FtsZ binding proteins in previous studies. As a result, it was largely neglected. However, structural models of the FtsZ–ZapA complex by AlphaFold 3 implied that the N‐terminal motif of FtsZ was involved in binding ZapA. Indeed, we found that mutations in this short motif significantly affected FtsZ interaction with ZapA in vivo and in vitro. Moreover, sequence alignment revealed that this motif is highly conserved in bacteria encoding ZapA, but not in those without ZapA, indicating a pivotal role of this motif in their interaction. A previous study found that a mutation in the groove of the ZapA dimer head (N60Y) abolished the interaction with FtsZ in vivo^36^. In the structural models, the N60 residue of ZapA interacts with the F2 residue of FtsZ. Thus, it is not surprising that mutating either one of these two residues strongly reduces or eliminates the interaction between FtsZ and ZapA. A recent Cryo‐EM structure of the ZapA tetramer–FtsZ filament complex shows that the ZapA dimer head binds to this N‐terminal motif of FtsZ^60^, further supporting our finding. Intriguingly, in this structure, ZapA tetramers do not bind to the junction between FtsZ subunits in a single filament. Also, each dimer head binds to two N‐terminal motifs, with each coming from an FtsZ molecule in an anti‐parallel double FtsZ filament^60^. This seems to be contrary to the current working model in which ZapA tetramers align FtsZ filaments into a parallel manner^46^. It is possible that the condition used for visualization of the ZapA tetramer–FtsZ filament complex was prone to the formation of anti‐parallel FtsZ filaments, allowing ZapA to bind to the N‐terminal motif of FtsZ but prevented it from binding to the junction between FtsZ subunits within the filaments. Future structural studies of ZapA and FtsZ in the absence of the N‐terminal motif of FtsZ are necessary to obtain a clearer picture of the interaction interface between ZapA and FtsZ filaments.

Interestingly, several mutations conferring resistance to ZapA overexpression are buried inside the FtsZ molecule and away from the deduced binding sites. Characterization of one of these mutations, V128I, showed that it reduced the interaction between FtsZ and ZapA. How could such mutations affect the interaction between FtsZ and ZapA if they are not directly involved in the interaction? It is well documented that there are substantial conformational changes in the polymerization interface and nearby regions during FtsZ polymerization and depolymerization^4^, ^57^, ^58^. It is possible that the isolated mutations cause an alteration of the conformation of FtsZ within the filament, even modestly, such that the binding affinity for ZapA is reduced drastically. Nonetheless, ZapA could still bind to the N‐terminal motif so that it still localized to the midcell in cells expressing the V128I mutant as shown in Figure 9. Further studies to look at the effects of the mutations on the polymerization interface and dynamics of FtsZ are necessary to reveal the molecular basis of these mutations.

**Figure 10:**
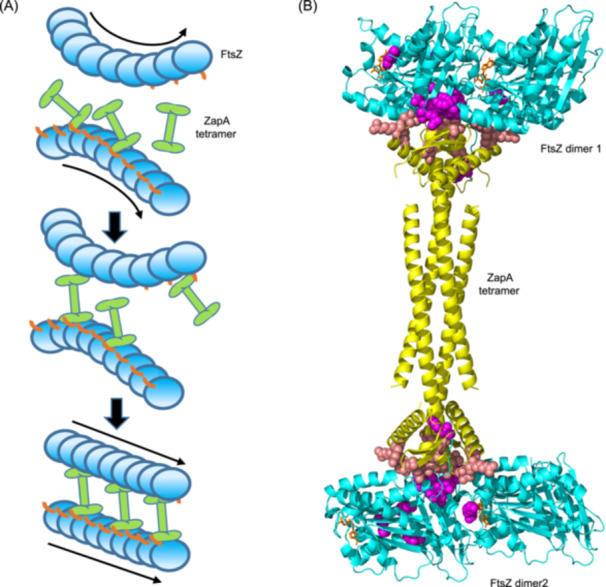
A proposed model for the mechanism of ZapA‐mediated straightening and crosslinking of FtsZ filaments. (A) A diagram depicting the mechanism of ZapA. ZapA tetramers bind to the N‐terminal tail of FtsZ to associate with curved FtsZ filaments. Subsequently, each dimer head of a ZapA tetramer binds to the junction of FtsZ subunits in a filament, functioning as a staple to straighten the longitudinal interaction between FtsZ subunits. Since a ZapA tetramer is bipolar with a triple twofold symmetry, the two dimer heads can crosslink two adjacent parallel FtsZ filaments that rotate 180° to each other. In this way, ZapA tetramers straighten and crosslink FtsZ filaments simultaneously to facilitate their organization into the Z ring. The linker and the conserved C‐terminal peptide of FtsZ are omitted for simplicity. The treadmilling directions of FtsZ filaments are indicated by the arrows. (B) A docking model for the FtsZ–ZapA complex. FtsZ dimers from the filament structure of *K. pneumoniae* FtsZ (PDB#: 8IBN) and the ZapA tetramer in complex with the N‐terminal motif of FtsZ (predicted by AlphaFold 3) were used to generate the model by HDOCK. The ZapA tetramer, colored yellow, binds to both the N‐terminal motif and the junction between FtsZ subunits in the filament. Residues important for FtsZ binding to ZapA are colored magenta, whereas those important for ZapA binding to FtsZ are colored pink. GTP is colored brown and shown as a stick.

The dual binding mode between ZapA and FtsZ filaments suggests that the ZapA tetramer grabs the N‐terminal tail of an FtsZ subunit to bind to a filament and positions its dimer heads into the junction between FtsZ subunits in the filament. This binding, analogous to a constructive staple, could stabilize the junction between FtsZ subunits, leading to the straightening of the filaments. Meanwhile, as each dimer head can bind to a filament, the ZapA tetramer is able to crosslink a pair of parallel FtsZ filaments that are related a 180° rotation around the Z‐axis (Figure 10A). To validate this model, we used the HDOCK docking algorithm^61^, ^62^ to predict the FtsZ–ZapA complex based on the filament structure of *K. pneumoniae* FtsZ and the AlphaFold 3 structural model of the ZapA tetramer in complex with the N‐terminal motif of FtsZ. By constraining the distance between residues 10 and 11 of FtsZ and applying additional constraints based on known mutation sites on FtsZ that affect its interaction with ZapA, we obtained a model of the FtsZ–ZapA complex compatible with our finding. In this model, each dimer head of a ZapA tetramer binds to one N‐terminal motif of FtsZ and the junction between FtsZ subunits so that the ZapA tetramer can crosslink two FtsZ dimers that are not entirely parallel (Figure 10B). It is known that the coiled‐coil domains of ZapA can undergo conformational change, resulting in rotation of the dimer heads. As a result, it is possible that the binding between FtsZ and the dimer heads of ZapA induces conformational changes in both FtsZ filaments and ZapA, resulting in the relative movement of the dimer head and aligning FtsZ filaments in parallel. Moreover, FtsZ filaments have been reported to assemble into inherently curved and twisted filaments^16^, ^63^, ^64^, ^65^; ZapA tetramers are able to crosslink multiple parallel twisted FtsZ filaments along their length into large bundles as observed under the electron microscope.

How could ZapA straighten and crosslink FtsZ filaments without affecting the dynamics of individual filaments? It is well documented that FtsZ filaments are semiflexible and intrinsically curved because the relative positions of adjacent FtsZ subunits in the filament are dynamically governed by its GTPase activity^66^, ^67^, ^68^, ^69^. The nucleotide (GTP/GDP) between FtsZ subunits governs the interaction strength and conformation of the polymerization interface (junction), with GTP stabilizing the interaction, while GTP hydrolysis weakens it. Since FtsZ filaments grow from one end and shrink from the opposite end following GTP hydrolysis, FtsZ subunits near the growing end are likely bound with GTP, whereas those near the shrinking end are likely bound with GDP. This asymmetric distribution of GTP/GDP along the FtsZ filament indicates that the junctions near the growing end are tighter, while those near the shrinking end are weaker. ZapA may prefer junctions near the growing end more than those near the shrinking end. As the GTP is hydrolyzed, the configuration of the junction is altered, and ZapA dissociates from the filaments near the depolymerizing end of the filament. Thus, by coupling its binding to FtsZ with the polymerization dynamics and structural flexibility of the FtsZ filaments, ZapA can organize FtsZ filaments dynamically without affecting the treadmilling speed (GTPase activity) or filament length.

## MATERIALS AND METHODS

### Bacterial strains, plasmids, and growth conditions

Strains were grown in Luria–Bertani (LB) medium (1% tryptone, 0.5% yeast extract, 0.5% NaCl, and pH was adjusted to 7.5 by adding 1 M NaOH solution) at indicated temperatures. Antibiotics were used at the following concentrations when necessary: 100 μg/ml ampicillin; 25 μg/ml spectinomycin; 25 μg/ml kanamycin; 12.5 μg/ml tetracycline; and 15 μg/ml chloramphenicol. The bacterial strains, plasmids, and primers used in this study are listed in Tables S3–S5. Construction of strains and plasmids is described in detail in the Supplemental Information.

### Creation of the FtsZ mutant library and selection for ZapA overexpression resistant *ftsZ* mutations

The coding region of *ftsZ* was mutagenized by PCR random mutagenesis using pBANG112 as the template and primers of 5′‐CCG**GAATTC**TTCGCGGTAAATACC‐3′ and 5′‐GCA**TCGGC**CGGGAAATCTAC‐3′. The purified PCR fragments and pBANG112 were digested with *Eco*RI and *Eag*I and then ligated. The ligation product was transformed into JS238 and transformants were selected at 37°C on LB plates with ampicillin. All colonies were pooled together and plasmids were extracted from the pooled culture, saved as the FtsZ mutant library. To select for the ZapA overexpression resistant FtsZ mutants, the FtsZ mutant library pBANG112^M^ and plasmid pSD319 were co‐transformed into S17/pKD3C. Colonies resistant to ZapA overexpression were selected at 42°C on plates containing ampicillin, kanamycin, and 30 μM IPTG. Plasmids were isolated from the ZapA‐resistant colonies and the *ftsZ* gene was sequenced to identify the mutations.

### Complementation test

Plasmid pBANG112 or its derivatives were used for complementation tests of the FtsZ depletion strain. Plasmids carrying the *ftsZ* alleles were transduced into the depletion strain S17/pKD3C (*ftsZ* ^*0*^/pSC101^*ts*^, *ftsZ*) at 30°C. Since pKD3C cannot replicate at temperatures above 37°C, the complementation tests were conducted at 42°C by a spot test.

### Fluorescence microscopy

Phase contrast and epifluorescence images were collected on an Olympus BX53 upright microscope with a Retiga R1 camera from QImaging, a CoolLED pE‐4000 light source, and a U Plan XApochromat phase contrast objective lens (100 × 1.45 numerical aperture [NA], oil immersion). Green and red fluorescence images were acquired using the Chroma EGFP filter set 49002 and the mCherry/Texas Red filter set 49008, respectively. Cells were immobilized on 2% agarose pads for microscopy.

#### Co‐localization of ZipA‐mCherry with GFP‐FtsI

Overnight culture of CYa35 (*zipA‐mCherry‐spc*, *ftsZ* ^*0*^/pACYC::*ftsZ*, and pEXT22, P_*tac*_::*zapA*) carrying plasmid pCY205 (pZH509, *gfp‐ftsI*, *cat*) was diluted 1:100 in fresh LB. After incubation at 37°C with shaking for 2.5 h, the culture was diluted 1:10 in fresh LB and then grown at 37°C. The cultures were grown for another 1.5 h with or without 500 μM IPTG. 2 μl of the cultures was spot on 2% agarose pads for photograph. The culture without IPTG was also treated with cephalexin to serve as a control. ZipA‐mCherry rings and GFP–FtsI rings in cells (– IPTG: *n* = 674, + IPTG: *n* = 171) were manually counted and their ratio of co‐localization was determined.

#### Fluorescent d‐amino acid labeling

Nascent peptidoglycan was labeled with HADA (7‐hydroxycoumarin‐3‐carboxylic acid‐D alanine), which was prepared in DMSO (25 mM) and stored at −20°C before use. A final concentration of 0.25 mM of HADA was used in all the related experiments. The cell cultures were incubated with HADA at 30°C for 1 min. The cells were then fixed with 2.6% paraformaldehyde and 0.04% pentadiol for 15 min on an ice bath. The cells were then collected by centrifugation at 12,000 rpm for 1 min, washed with 1× PBS, and resuspended into 1× PBS for subsequent imaging by fluorescence microscopy. In experiments to check the co‐localization of ZipA‐mCherry in the absence or presence of ZapA overexpression, the cell cultures of CYa35 (W3110, *ftsZ* ^*0*^ *zipA‐mCherry*/pACYC, *ftsZ* and pEXT22, P_*tac*_::*zapA*) were grown with or without 500 μM IPTG for 1 h, and incubated with HADA to label nascent PG. The culture without IPTG was treated with cephalexin to serve as a negative control. ZipA‐mCherry rings and HADA rings in cells were counted (− IPTG: *n* = 515; + IPTG: *n* = 230) and the co‐localization ratio was determined.

#### Localization of ZipA‐mCherry in cells expressing wild‐type or FtsZ mutants

An overnight culture of CYa35 (*ftsZ* ^*0*^, *zipA‐mCherry‐spc*/pACYC, *ftsZ *and pEXT22, P_*tac*_::*zapA*) was diluted 1:100 in fresh LB and grown at 37°C for 2.5 h. Then, the culture was diluted 1:10 in fresh LB with or without 500 μM IPTG and cultured to OD_600_ of ~0.5. 2 μl of the cultures was spotted on 2% agarose pads for photograph. Strains expressing FtsZ variants were observed similarly.

#### Localization of ZapA‐GFP in cells expressing wild‐type or FtsZ mutants

To examine ZapA–GFP localization, an overnight culture of CYb1 (*ftsZ* ^*0*^, zapA‐gfp‐cat, *zapB*::*kan*/pACYC, *ftsZ*) was grown in LB at 37°C to the exponential phase. The culture was then diluted 1:10 in fresh LB and cultured to an OD_600_ of ~0.5. The sample was spotted on 2% agarose pads for microscopy. Strains expressing FtsZ variants were observed similarly.

#### Time‐lapse imaging

To follow the dynamics of Z ring/structures more closely, CYm142 (W3110/pZH509, *ftsZ*‐linker5‐*mNG*) and CYm143 (W3110/pZH509, *ftsZ*‐linker5‐*mNG* and pEXT22, P_*tac*_::*zapA*) were grown to the log phase as described above and imaged using a High Intelligent and Sensitive SIM (HIS‐SIM) P‐104WT microscope equipped with an Apo 100×/1.5× Oil objective. Time‐lapse imaging of FtsZ‐mNG was performed at 10 s intervals over a 10‐min period, with excitation by a 488 nm laser, an exposure time of 60 ms, and an illumination intensity of 0.2 W/cm². Bacterial samples were mounted in confocal glass‐bottom dishes, and acquired images were subsequently processed using Fiji for movie production.

#### FtsZ treadmilling imaging

To determine the FtsZ treadmilling speed, images of FtsZ‐mNG were acquired as previously described using a Nikon inverted microscope equipped with a TELEDYNE CMOS (Prime BSI Express) camera and a Nikon 100× NA 1.49 TIRF objective^19^. FtsZ‐mNG expressed from P_*tac*_::*ftsZ‐mNG* was excited under the total internal reflection fluorescence (TIRF) mode. Images were acquired at 1 s intervals with an exposure time of 50 ms for 150 frames.

### Bacterial two‐hybrid analysis

To detect the FtsZ–ZapA interaction using the bacterial two‐hybrid assay, appropriate plasmid pairs encoding ZapA‐T18 and FtsZ^1–316^‐T25 or its derivatives containing FtsZ mutations were co‐transformed into BTH101. Single colonies were picked and resuspended in 1 ml of LB medium. 2.5 μl of each aliquot was spotted on LB plates supplemented with appropriate antibiotics, 40 μg/ml X‐gal, and 20 μM IPTG. Plates were incubated at 30°C for 12 h. Interactions between ZapA and FtsZ^1–316^ or their variants were tested similarly.

### In vivo BMOE crosslinking assays

Strains CYm141 (*ftsZ*::*cat zapA* < >*frt*/pACYC, *ftsZ* ^*N73C*^) or its derivatives were transformed with pCY173 (pEXT22, P_*tac*_::*zapA* ^*C19A,T50C*^‐*gfp*, *Kan* ^*r*^). Overnight cultures of transformed strains were diluted 1:100 into LB with antibiotics and inducer at the following concentration (pBANG112: 100 μg/ml ampicillin; pCY173: 100 μM IPTG and 25 μg/ml kanamycin). Cells were grown at 37°C to the exponential phase, and 2 ml cultures were harvested (10,000 rpm, 2 min), washed with 2 ml of PBSG (PBS with 0.1% Glycerine) twice, and then resuspended in 1 ml of PBSG with 5 mM EDTA. Cells were treated with 400 μM BMOE (Thermo Scientific) at 4°C for 15 min, with DMF alone serving as the negative control. Samples were treated with 2 μl of β‐mercaptoethanol to quench the BMOE reaction. Cells were collected (10,000 rpm for 5 min), lysed in 50 μl 1× SDS‐PAGE loading buffer containing 5% β‐mercaptoethanol, and boiled for 10 min before loading into 10% SDS‐PAGE gels. Proteins were transferred to polyvinylidene fluoride membranes and immunoblotted with rabbit antiserum against FtsZ and a goat anti‐rabbit antibody. The membranes were imaged under fluorescence mode in a ChemiDoc MP system (Bio‐Rad).

### Protein purification

An overnight culture of BL21/pLys harboring plasmid pE‐SUMO‐FtsZ (P_*T7*_::*his‐SUMO‐ftsZ*) was diluted 1:100 in fresh LB with antibiotics. After incubation at 37°C with shaking for 2 h, 500 μM IPTG was added. The culture was grown at 37°C for another 3 h and cells were collected by centrifugation at 8000 rpm for 10 min at 4°C. The cells were resuspended in 20 ml of lysis buffer (25 mM Tris‐HCl [pH 7.5], 300 mM NaCl, 0.1 mM DTT, 20 mM imidazole, and 5% glycerol) and cell debris was removed by centrifugation (10,000 rpm, 10 min) at 4°C. The supernatants were loaded onto pre‐equilibrated Ni‐NTA resin (Qiagen), and then the resin was washed twice with a high‐salt wash buffer (25 mM Tris‐HCl [pH 7.5], 500 mM NaCl, 0.1 mM DTT, 20 mM imidazole, and 5% glycerol). The bound protein was eluted with elution buffer (25 mM Tris‐HCl [pH 7.5], 500 mM NaCl, 0.1 mM DTT, 250 mM imidazole, and 5% glycerol) and analyzed by SDS‐PAGE. The fractions with large amounts of proteins were pooled together and dialyzed against the dialysis buffer (25 mM Tris‐HCl [pH 7.5], 300 mM NaCl, 0.1 mM DTT, and 5% glycerol) overnight and dialyzed against the fresh dialysis buffer for 6 h the next day. After dialysis, the His‐SUMO tag was cleaved by incubation with 6× His‐tagged SUMO protease (Ulp1) for 1 h at 30°C. The mixture was then passed through the pre‐equilibrated Ni‐NTA resin to remove the released tag and protease, while FtsZ was collected in the flow through. Purified FtsZ was then concentrated and stored at −80°C until use. All FtsZ mutant proteins were purified similarly.

H‐SUMO‐ZapA was expressed and purified from BL21 (DE3)/pLys cells containing pCY52 (P_*T7*_::*his‐SUMO‐ZapA*) similar to the purification of H‐SUMO‐FtsZ. The His‐SUMO tag of H‐SUMO‐ZapA was cleaved with purified 6× His‐tagged SUMO protease (Ulp1) as H‐SUMO‐FtsZ.

### GTPase assay

GTPase activities of FtsZ and its mutants were determined using the NADH‐coupled enzymatic assay at room temperature^54^. The reactions were performed in 200 µl volume using the FtsZ polymerization buffer (50 mM HEPES‐NaOH [pH 6.8], 50 mM KCl, 10 mM MgCl_2_), plus 1 mM PEP, and 1.2 mM NADH, 5 µl PK/LDH, and 2.5 mM GTP. 2.5 µM FtsZ or its mutants were added for the assay. NADH depletion is coupled with GTP hydrolysis; thus, the reactions were continuously monitored for NADH absorbance at 340 nm for 30 min. Reaction rates were determined by plotting the OD_340_ of samples against time using Prism software and calculated using the Beer–Lambert law (*A *= ε *×* *c *× *l*).

### Pull‐down assay

The pull‐down assay was performed at 4°C. H‐SUMO‐ZapA (6 μM) and FtsZ (2 μM) were mixed in a total volume of 500 μl Pol buffer (50 mM HEPES NaOH [pH 6.8], 50 mM KCl, and 10 mM MgCl_2_). The mixtures were incubated at room temperature for 10 min and then loaded into a column with 200 μl pre‐equilibrated Ni‐NTA agarose. After incubation on ice for 5 min, the mixture was passed through the column by centrifugation at 2000 rpm for 30 s at 4°C. The column was then washed with 500 μl of wash buffer (25 mM Tris‐HCl [pH 7.5], 500 mM NaCl, 0.1 mM DTT, 20 mM imidazole, and 5% glycerol) twice. Proteins bound to the Ni‐NTA beads were eluted with 500 μl of elution buffer (25 mM Tris‐HCl [pH 7.5], 500 mM NaCl, 0.1 mM DTT, 250 mM imidazole, and 5% glycerol). All fractions were collected during the procedure and analyzed by SDS‐PAGE. All FtsZ mutant proteins were tested similarly.

### Sedimentation assay and electron microscopy

FtsZ or its variants (5 µM) were tested for polymerization before the sedimentation assay. The reactions were performed in 50 µl of Pol buffer at room temperature. FtsZ or its variants (5 µM) were mixed with or without 10 mM CaCl_2_ at room temperature for 5 min. After the addition of 2.5 mM GTP or GDP, the reactions were kept at room temperature for 5 min and then subjected to ultracentrifugation at 100,000 rpm for 15 min at 25°C in a Beckman Optima Max XP centrifuge with a TAL130 rotor (Beckman Coulter, Inc.). Supernatants and pellets were then analyzed by SDS‐PAGE. In experiments in which ZapA was added, FtsZ or its variants (5 µM) were mixed with or without untagged ZapA (5 µM) at room temperature for 5 min before the addition of 2.5 mM GTP. After 5 min at room temperature, the samples were handled as described for the sedimentation assay.

To visualize the effect of ZapA on the filaments of FtsZ or its mutants by electron microscopy, FtsZ or the mutant proteins (2.5 µM) and untagged ZapA (2.5 µM) were mixed together and 1 mM GTP was added to initiate polymerization. After a 10 min incubation at room temperature, 15 µl samples were loaded onto glow‐discharged grids. The excess solution was then blotted away after another 5 min, and the grid was stained with 15 µl of 1% uranyl acetate for 1 min and then blotted away. The grids were air‐dried for more than 12 h and imaged with a JEM‐1400Plus transmission electron microscope. When comparing the effect of ZapA on wild‐type FtsZ and FtsZ^F2A^ filaments, the final concentration of proteins was lowered to 1 µM because FtsZ^F2A^ tends to assemble into bundles by itself at higher concentrations.

### FtsZ filaments' treadmilling speed and orientation analysis

The FtsZ filaments (or clusters) with obverse movements along a certain orientation were selected and segmented manually. The selected frames of the movie were used for the maximum projection of intensity (MPI) using Fiji^70^. The angle (*θ*) between the orientation of the fluorescence line in the MPI image and the long axis of the cell was measured using the Angle Tool in Fiji. The treadmilling speed was measured as previously described^19^. Briefly, drift correction was applied using the HyperStackReg plugin in Fiji if needed. Every image frame in the time‐lapse fluorescence movie (in TIRF mode) was denoised using the PureDenoise plugin in Fiji (developed by Florian Luisier at the Biomedical Imaging Group (BIG), EPFL, Switzerland). The process used a global estimation and performed 10 iterations with a moving average over a 3‐frame window. Next, each movie was enlarged to a pixel size of 21.7 nm using the bicubic interpolation method in Fiji. Subsequently, every movie was processed using the kymograph plugin (https://github.com/remiberthoz/imagej-live-kymographer) with a 3‐frame average for smoothing. Kymographs were then created using the kymograph plugin from a line 13 pixels wide (~280 nm) across the Z ring. The treadmilling speeds from both the leading and trailing edges were calculated. The box diagram plotted in OriginLab software was based on data from three independent experiments.

### Docking of FtsZ and the ZapA tetramer

To validate our model for the interaction between FtsZ and ZapA, we used the HDOCK docking algorithm using the available FtsZ filament structure and AlphaFold 3 generated structural models. Since the AlphaFold3 server cannot accurately predict the complex structure of FtsZ and ZapA, and the ipTM value is quite low, the results are clearly unreliable. However, based on the predictions, the N‐terminal of FtsZ is likely to interact with ZapA. Therefore, we used the AlphaFold3 server to predict the structure of ZapA in complex with the N‐terminal of FtsZ (residues 1–10). The resulting model showed an ipTM value of 0.57 between the N‐terminal of FtsZ and ZapA, indicating a certain level of reliability. Subsequently, we used the HDOCK docking algorithm to predict the interaction structure between the polymerization domain of FtsZ (residues 11–316) and the FtsZ N‐terminal–ZapA complex. During the docking process, we constrained the distance between residues 10 and 11 of FtsZ to ensure that the N‐terminal motif of FtsZ connects seamlessly with the remaining portion in the docking model. Also, our data showed that mutations at residues 66, 140, 141, 214, 215, 218, 219, 258, 289, and 291 of FtsZ, which are clustered in a specific region, weaken its interaction with ZapA. Therefore, we applied these residues as constraints to filter the generated docking models, eliminating those that did not satisfy the constraints. Subsequently, we manually inspected the remaining docking models, selecting those in which the head domain of ZapA interacts well with the constrained region of FtsZ. The final selected model was used for further analysis.

## AUTHOR CONTRIBUTIONS

**Yuanyuan Cui:** Conceptualization; investigation; writing—original draft. **Han Gong:** Investigation; Data curation; methodology. **Di Yan:** Investigation; Data curation; methodology. **Hao Li:** Investigation; Data curation; methodology. **Wenjie Yang:** Data curation; methodology. **Ying Li:** Methodology. **Xiangdong Chen:** Resources; supervision. **Joe Lutkenhaus:** Resources; Writing—review and editing. **Sheng‐You Huang:** Resources; Supervision; Funding acquisition. **Xinxing Yang:** Methodology; Data curation; Resources; Supervision; Funding acquisition. **Shishen Du:** Conceptualization; Data curation; Funding acquisition; supervision; writing—review and editing.

## ETHICS STATEMENT

This study did not involve any human participants or animal subjects.

## CONFLICT OF INTERESTS

The authors declare no conflict of interests.

## Supporting information

SI 2025‐05‐23.

ZapA video.

## Data Availability

All data related to this study are presented in the main text or the Supplemental Information. Strains and plasmids are available from the corresponding author upon reasonable request with material transfer agreement.

## References

[1] Bi E , Lutkenhaus J . FtsZ ring structure associated with division in *Escherichia coli* . Nature. 1991;354:161–164. 1944597 10.1038/354161a0

[2] Yang X , Lyu Z , Miguel A , McQuillen R , Huang KC , Xiao J . GTPase activity‐coupled treadmilling of the bacterial tubulin FtsZ organizes septal cell wall synthesis. Science. 2017;355:744–747. 28209899 10.1126/science.aak9995 PMC5851775

[3] Bisson‐Filho AW , Hsu YP , Squyres GR , Kuru E , Wu F , Jukes C , et al. Treadmilling by FtsZ filaments drives peptidoglycan synthesis and bacterial cell division. Science. 2017;355:739–743. 28209898 10.1126/science.aak9973 PMC5485650

[4] McQuillen R , Xiao J . Insights into the structure, function, and dynamics of the bacterial cytokinetic FtsZ‐ring. Annu Rev Biophys. 2020;49:309–341. 32092282 10.1146/annurev-biophys-121219-081703 PMC8610174

[5] Cameron TA , Margolin W . Insights into the assembly and regulation of the bacterial divisome. Nat Rev Microbiol. 2024;22:33–45. 37524757 10.1038/s41579-023-00942-x PMC11102604

[6] Thanedar S , Margolin W . FtsZ exhibits rapid movement and oscillation waves in helix‐like patterns in *Escherichia coli* . Curr Biol. 2004;14:1167–1173. 15242613 10.1016/j.cub.2004.06.048 PMC4757587

[7] Squyres GR , Holmes MJ , Barger SR , Pennycook BR , Ryan J , Yan VT , et al. Single‐molecule imaging reveals that Z‐ring condensation is essential for cell division in *Bacillus subtilis* . Nat Microbiol. 2021;6:553–562. 33737746 10.1038/s41564-021-00878-z PMC8085161

[8] Whitley KD , Jukes C , Tregidgo N , Karinou E , Almada P , Cesbron Y , et al. FtsZ treadmilling is essential for Z‐ring condensation and septal constriction initiation in *Bacillus subtilis* cell division. Nat Commun. 2021;12:2448. 33907196 10.1038/s41467-021-22526-0 PMC8079713

[9] Fu G , Huang T , Buss J , Coltharp C , Hensel Z , Xiao J . In vivo structure of the *E. coli* FtsZ‐ring revealed by photoactivated localization microscopy (PALM). PLoS One. 2010;5:e12682. 10.1371/journal.pone.0012680 PMC2938336 20856929

[10] Strauss MP , Liew ATF , Turnbull L , Whitchurch CB , Monahan LG , Harry EJ . 3D‐SIM super resolution microscopy reveals a bead‐like arrangement for FtsZ and the division machinery: implications for triggering cytokinesis. PLoS Biol. 2012;10:e1001389. 22984350 10.1371/journal.pbio.1001389 PMC3439403

[11] Biteen JS , Goley ED , Shapiro L , Moerner WE . Three‐dimensional super‐resolution imaging of the midplane protein FtsZ in live *Caulobacter crescentus* cells using astigmatism. Chemphyschem. 2012;13:1007–1012. 22262316 10.1002/cphc.201100686 PMC3712621

[12] Yang X , McQuillen R , Lyu Z , Phillips‐Mason P , De La Cruz A , McCausland JW , et al. A two‐track model for the spatiotemporal coordination of bacterial septal cell wall synthesis revealed by single‐molecule imaging of FtsW. Nat Microbiol. 2021;6:584–593. 33495624 10.1038/s41564-020-00853-0 PMC8085133

[13] Mahone CR , Payne IP , Lyu Z , McCausland JW , Barrows JM , Xiao J , et al. Integration of cell wall synthesis and chromosome segregation during cell division in *Caulobacter* . J Cell Biol. 2024;223:e202211026. 38015166 10.1083/jcb.202211026 PMC10683668

[14] Osawa M , Anderson DE , Erickson HP . Reconstitution of contractile FtsZ rings in liposomes. Science. 2008;320:792–794. 18420899 10.1126/science.1154520 PMC2645864

[15] Loose M , Mitchison TJ . The bacterial cell division proteins FtsA and FtsZ self‐organize into dynamic cytoskeletal patterns. Nat Cell Biol. 2014;16:38–46. 24316672 10.1038/ncb2885 PMC4019675

[16] Ramirez‐Diaz DA , García‐Soriano DA , Raso A , Mücksch J , Feingold M , Rivas G , et al. Treadmilling analysis reveals new insights into dynamic FtsZ ring architecture. PLoS Biol. 2018;16:e2004845. 29775478 10.1371/journal.pbio.2004845 PMC5979038

[17] Kohyama S , Merino‐Salomón A , Schwille P . In vitro assembly, positioning and contraction of a division ring in minimal cells. Nat Commun. 2022;13:6098. 36243816 10.1038/s41467-022-33679-x PMC9569390

[18] Huang KH , Durand‐Heredia J , Janakiraman A . FtsZ ring stability: of bundles, tubules, crosslinks, and curves. J Bacteriol. 2013;195:1859–1868. 23457247 10.1128/JB.02157-12 PMC3624584

[19] Gong H , Yan D , Cui Y , Li Y , Yang J , Yang W , et al. The divisome is a self‐enhancing machine in *Escherichia coli* and *Caulobacter crescentus* . Nat Commun. 2024;15:8198. 39294118 10.1038/s41467-024-52217-5 PMC11410940

[20] Gueiros‐Filho FJ , Losick R . A widely conserved bacterial cell division protein that promotes assembly of the tubulin‐like protein FtsZ. Genes Dev. 2002;16:2544–2556. 12368265 10.1101/gad.1014102 PMC187447

[21] Buss J , Coltharp C , Huang T , Pohlmeyer C , Wang SC , Hatem C , et al. In vivo organization of the FtsZ‐ring by ZapA and ZapB revealed by quantitative super‐resolution microscopy. Mol Microbiol. 2013;89:1099–1120. 23859153 10.1111/mmi.12331 PMC3894617

[22] Durand‐Heredia JM , Yu HH , De Carlo S , Lesser CF , Janakiraman A . Identification and characterization of ZapC, a stabilizer of the FtsZ ring in *Escherichia coli* . J Bacteriol. 2011;193:1405–1413. 21216995 10.1128/JB.01258-10 PMC3067613

[23] Durand‐Heredia J , Rivkin E , Fan G , Morales J , Janakiraman A . Identification of ZapD as a cell division factor that promotes the assembly of FtsZ in *Escherichia coli* . J Bacteriol. 2012;194:3189–3198. 22505682 10.1128/JB.00176-12 PMC3370873

[24] Hale CA , Shiomi D , Liu B , Bernhardt TG , Margolin W , Niki H , et al. Identification of *Escherichia coli* ZapC (YcbW) as a component of the division apparatus that binds and bundles FtsZ polymers. J Bacteriol. 2011;193:1393–1404. 21216997 10.1128/JB.01245-10 PMC3067623

[25] Marteyn BS , Karimova G , Fenton AK , Gazi AD , West N , Touqui L , et al. ZapE is a novel cell division protein interacting with FtsZ and modulating the Z‐ring dynamics. mBio. 2014;5:e00022‐14. 24595368 10.1128/mBio.00022-14 PMC3958796

[26] Ebersbach G , Galli E , Møller‐Jensen J , Löwe J , Gerdes K . Novel coiled‐coil cell division factor ZapB stimulates Z ring assembly and cell division. Mol Microbiol. 2008;68:720–735. 18394147 10.1111/j.1365-2958.2008.06190.x

[27] Galli E , Gerdes K . Spatial resolution of two bacterial cell division proteins: ZapA recruits ZapB to the inner face of the Z‐ring. Mol Microbiol. 2010;76:1514–1526. 20487275 10.1111/j.1365-2958.2010.07183.x

[28] Woldemeskel SA , McQuillen R , Hessel AM , Xiao J , Goley ED . A conserved coiled‐coil protein pair focuses the cytokinetic Z‐ring in *Caulobacter crescentus* . Mol Microbiol. 2017;105:721–740. 28613431 10.1111/mmi.13731 PMC5570653

[29] Perez AJ , Villicana JB , Tsui HCT , Danforth ML , Benedet M , Massidda O , et al. FtsZ‐ring regulation and cell division are mediated by essential EzrA and accessory proteins ZapA and ZapJ in *Streptococcus pneumoniae* . Front Microbiol. 2021;12:780864. 34938281 10.3389/fmicb.2021.780864 PMC8687745

[30] Krupka M , Rowlett VW , Morado D , Vitrac H , Schoenemann K , Liu J , et al. *Escherichia coli* FtsA forms lipid‐bound minirings that antagonize lateral interactions between FtsZ protofilaments. Nat Commun. 2017;8:15957. 28695917 10.1038/ncomms15957 PMC5508204

[31] Krupka M , Sobrinos‐Sanguino M , Jiménez M , Rivas G , Margolin W . *Escherichia coli* ZipA organizes FtsZ polymers into dynamic ring‐like protofilament structures. mBio. 2018;9:e01008‐18. 29921670 10.1128/mBio.01008-18 PMC6016244

[32] Espéli O , Borne R , Dupaigne P , Thiel A , Gigant E , Mercier R , et al. A MatP‐divisome interaction coordinates chromosome segregation with cell division in *E. coli* . EMBO J. 2012;31:3198–3211. 22580828 10.1038/emboj.2012.128 PMC3400007

[33] Bailey MW , Bisicchia P , Warren BT , Sherratt DJ , Männik J . Evidence for divisome localization mechanisms independent of the Min system and SlmA in *Escherichia coli* . PLoS Genet. 2014;10:e1004504. 25101671 10.1371/journal.pgen.1004504 PMC4125044

[34] Buss J , Coltharp C , Shtengel G , Yang X , Hess H , Xiao J . A multi‐layered protein network stabilizes the *Escherichia coli* FtsZ‐ring and modulates constriction dynamics. PLoS Genet. 2015;11:e1005128. 25848771 10.1371/journal.pgen.1005128 PMC4388696

[35] Mercier R , Petit MA , Schbath S , Robin S , El Karoui M , Boccard F , et al. The MatP/*matS* site‐specific system organizes the terminus region of the *E. coli* chromosome into a macrodomain. Cell. 2008;135:475–485. 18984159 10.1016/j.cell.2008.08.031

[36] Buss JA , Peters NT , Xiao J , Bernhardt TG . ZapA and ZapB form an FtsZ‐independent structure at midcell. Mol Microbiol. 2017;104:652–663. 28249098 10.1111/mmi.13655 PMC5426985

[37] Du S , Lutkenhaus J . The N‐succinyl‐l,l‐diaminopimelic acid desuccinylase DapE acts through ZapB to promote septum formation in *Escherichia coli* . Mol Microbiol. 2017;105:326–345. 28470834 10.1111/mmi.13703 PMC5517081

[38] Ozaki S , Jenal U , Katayama T . Novel divisome‐associated protein spatially coupling the Z‐ring with the chromosomal replication terminus in *Caulobacter crescentus* . mBio. 2020;11:e00487‐20. 32345642 10.1128/mBio.00487-20 PMC7188993

[39] Ozaki S , Wakasugi Y , Katayama T . Z‐ring‐associated proteins regulate clustering of the replication terminus‐binding protein ZapT in *Caulobacter crescentus* . mBio. 2021;12:e02196‐20. 10.1128/mBio.02196-20 PMC7858052 33500340

[40] Low HH , Moncrieffe MC , Löwe J . The crystal structure of ZapA and its modulation of FtsZ polymerisation. J Mol Biol. 2004;341:839–852. 15288790 10.1016/j.jmb.2004.05.031

[41] Roach EJ , Kimber MS , Khursigara CM . Crystal structure and site‐directed mutational analysis reveals key residues involved in *Escherichia coli* ZapA function. J Biol Chem. 2014;289:23276–23286. 25002581 10.1074/jbc.M114.561928 PMC4156065

[42] Pacheco‐Gómez R , Cheng X , Hicks MR , Smith CJI , Roper DI , Addinall S , et al. Tetramerization of ZapA is required for FtsZ bundling. Biochem J. 2013;449:795–802. 23098212 10.1042/BJ20120140

[43] Meiresonne NY , den Blaauwen T . The in vitro non‐tetramerizing ZapA(I83E) mutant is unable to recruit ZapB to the division plane in vivo in *Escherichia coli* . Int J Mol Sci. 2020;21:3130. 32365468 10.3390/ijms21093130 PMC7246612

[44] Roseboom W , Nazir MG , Meiresonne NY , Mohammadi T , Verheul J , Buncherd H , et al. Mapping the contact sites of the *Escherichia coli* division‐initiating proteins FtsZ and ZapA by BAMG cross‐linking and site‐directed mutagenesis. Int J Mol Sci. 2018;19:2928. 30261644 10.3390/ijms19102928 PMC6213154

[45] Small E , Marrington R , Rodger A , Scott DJ , Sloan K , Roper D , et al. FtsZ polymer‐bundling by the *Escherichia coli* ZapA orthologue, YgfE, involves a conformational change in bound GTP. J Mol Biol. 2007;369:210–221. 17428494 10.1016/j.jmb.2007.03.025

[46] Caldas P , López‐Pelegrín M , Pearce DJG , Budanur NB , Brugués J , Loose M . Cooperative ordering of treadmilling filaments in cytoskeletal networks of FtsZ and its crosslinker ZapA. Nat Commun. 2019;10:5744. 31848350 10.1038/s41467-019-13702-4 PMC6917738

[47] Vanhille‐Campos C , Whitley KD , Radler P , Loose M , Holden S , Šarić A . Self‐organization of mortal filaments and its role in bacterial division ring formation. Nat Phys. 2024;20:1670–1678. 39416851 10.1038/s41567-024-02597-8 PMC11473364

[48] Galli E , Gerdes K . FtsZ‐ZapA‐ZapB interactome of *Escherichia coli* . J Bacteriol. 2012;194:292–302. 22056926 10.1128/JB.05821-11 PMC3256659

[49] Shen B , Lutkenhaus J . The conserved C‐terminal tail of FtsZ is required for the septal localization and division inhibitory activity of MinC(C)/MinD. Mol Microbiol. 2009;72:410–424. 19415799 10.1111/j.1365-2958.2009.06651.x PMC2759774

[50] Du S , Lutkenhaus J . SlmA antagonism of FtsZ assembly employs a two‐pronged mechanism like MinCD. PLoS Genet. 2014;10:e1004460. 25078077 10.1371/journal.pgen.1004460 PMC4117426

[51] Schumacher MA , Ohashi T , Corbin L , Erickson HP . High‐resolution crystal structures of *Escherichia coli* FtsZ bound to GDP and GTP. Acta Crystallogr F Struct Biol Commun. 2020;76:94–102. 32039891 10.1107/S2053230X20001132 PMC7010359

[52] Fujita J , Amesaka H , Yoshizawa T , Hibino K , Kamimura N , Kuroda N , et al. Structures of a FtsZ single protofilament and a double‐helical tube in complex with a monobody. Nat Commun. 2023;14:4073. 37429870 10.1038/s41467-023-39807-5 PMC10333351

[53] Datsenko KA , Wanner BL . One‐step inactivation of chromosomal genes in *Escherichia coli* K‐12 using PCR products. Proc Natl Acad Sci USA. 2000;97:6640–6645. 10829079 10.1073/pnas.120163297 PMC18686

[54] Ingerman E , Nunnari J . A continuous, regenerative coupled GTPase assay for dynamin‐related proteins. Methods Enzymol. 2005;404:611–619. 16413304 10.1016/S0076-6879(05)04053-X

[55] Karimova G , Pidoux J , Ullmann A , Ladant D . A bacterial two‐hybrid system based on a reconstituted signal transduction pathway. Proc Natl Acad Sci USA. 1998;95:5752–5756. 9576956 10.1073/pnas.95.10.5752 PMC20451

[56] Li Y , Hsin J , Zhao L , Cheng Y , Shang W , Huang KC , et al. FtsZ protofilaments use a hinge‐opening mechanism for constrictive force generation. Science. 2013;341:392–395. 23888039 10.1126/science.1239248 PMC3816583

[57] Du S , Pichoff S , Kruse K , Lutkenhaus J . FtsZ filaments have the opposite kinetic polarity of microtubules. Proc Natl Acad Sci USA. 2018;115:10768–10773. 30275322 10.1073/pnas.1811919115 PMC6196554

[58] Abramson J , Adler J , Dunger J , Evans R , Green T , Pritzel A , et al. Accurate structure prediction of biomolecular interactions with AlphaFold 3. Nature. 2024;630:493–500. 38718835 10.1038/s41586-024-07487-w PMC11168924

[59] Dajkovic A , Pichoff S , Lutkenhaus J , Wirtz D . Cross‐linking FtsZ polymers into coherent Z rings. Mol Microbiol. 2010;78:651–668. 20969647 10.1111/j.1365-2958.2010.07352.x

[60] Fujita J , Hibino K , Kagoshima G , Kamimura N , Kato Y , Uehara R , et al. Structural basis for the interaction between the bacterial cell division proteins FtsZ and ZapA. Nat Commun. 2025;16: 5985. 40593603 10.1038/s41467-025-60940-w PMC12216130

[61] Yan Y , Zhang D , Zhou P , Li B , Huang SY . HDOCK: a web server for protein‐protein and protein‐DNA/RNA docking based on a hybrid strategy. Nucleic Acids Res. 2017;45:W365–W373. 28521030 10.1093/nar/gkx407 PMC5793843

[62] Yan Y , Tao H , He J , Huang SY . The HDOCK server for integrated protein‐protein docking. Nat Protoc. 2020;15:1829–1852. 32269383 10.1038/s41596-020-0312-x

[63] Arumugam S , Chwastek G , Fischer‐Friedrich E , Ehrig C , Mönch I , Schwille P . Surface topology engineering of membranes for the mechanical investigation of the tubulin homologue FtsZ. Angew Chem Int Ed. 2012;51:11858–11862. 10.1002/anie.201204332 22936525

[64] González de Prado Salas P , Hörger I , Martín‐García F , Mendieta J , Alonso Á , Encinar M , et al. Torsion and curvature of FtsZ filaments. Soft Matter. 2014;10:1977–1986. 24652404 10.1039/c3sm52516c

[65] Lv D , Li J , Ye S . The assembly switch mechanism of FtsZ filament revealed by all‐atom molecular dynamics simulations and coarse‐grained models. Front Microbiol. 2021;12:639883. 33859629 10.3389/fmicb.2021.639883 PMC8042166

[66] Erickson HP . Modeling the physics of FtsZ assembly and force generation. Proc Natl Acad Sci USA. 2009;106:9238–9243. 19478069 10.1073/pnas.0902258106 PMC2695047

[67] Erickson HP , Anderson DE , Osawa M . FtsZ in bacterial cytokinesis: cytoskeleton and force generator all in one. Microbiol Mol Biol Rev. 2010;74:504–528. 21119015 10.1128/MMBR.00021-10 PMC3008173

[68] Hsin J , Gopinathan A , Huang KC . Nucleotide‐dependent conformations of FtsZ dimers and force generation observed through molecular dynamics simulations. Proc Natl Acad Sci USA. 2012;109:9432–9437. 22647609 10.1073/pnas.1120761109 PMC3386107

[69] Knapp BD , Shi H , Huang KC . Complex state transitions of the bacterial cell division protein FtsZ. Mol Biol Cell. 2024;35:ar130. 39083352 10.1091/mbc.E23-11-0446 PMC11481701

[70] Schindelin J , Arganda‐Carreras I , Frise E , Kaynig V , Longair M , Pietzsch T , et al. Fiji: an open‐source platform for biological‐image analysis. Nat Methods. 2012;9:676–682. 22743772 10.1038/nmeth.2019 PMC3855844

