# Supplementary material for: ZapA uses a two‐pronged mechanism to facilitate Z ring formation in Escherichia coli
Source: mLife. 2025 Dec 21;4(6):604–24. doi: 10.1002/mlf2.70037 (PMC12754632; doi:10.1002/mlf2.70037)
Supplement: Supplementary file 1 — SI 2025‐05‐23. [file MLF2-4-604-s001.pdf]

Supplementary Information For:

ZapA employs a two-pronged mechanism to facilitate Z ring formation in *Escherichia coli*

Authors

Yuanyuan Cui[1, 2, 3], Han Gong[1, 2, 3], Di Yan[4], Hao Li[5], Wenjie Yang[1, 2, 3] , Ying Li[1, 2, 3], Xiangdong Chen[6], Joe Lutkenhaus[7], Sheng-You Huang[5], Xinxing Yang[4], and Shishen Du[1, 2, 3]*

Affiliation

1 State Key Laboratory of Metabolism and Regulation in Complex Organisms, College of Life Sciences, Wuhan University, Wuhan, China
2 Hubei Key Laboratory of Cell Homeostasis, College of Life Sciences, Wuhan University, Wuhan, Hubei, China
3 Key Laboratory of Polar Environment Monitoring and Public Governance (Wuhan University), Ministry of Education, China
4 Division of Life Sciences and Medicine, University of Science and Technology of China, Hefei, Anhui, China
5 School of Physics, Huazhong University of Science and Technology, Wuhan, Hubei, China
6 State Key Laboratory of Virology, College of Life Sciences, Wuhan University, Wuhan, Hubei, China
7 Department of Microbiology, Molecular Genetics and Immunology, University of Kansas Medical Center, Kansas City, Kansas, USA

*** To whom correspondence should be addressed:**

Shishen Du
State Key Laboratory of Metabolism and Regulation in Complex Organisms,
College of Life Sciences, Wuhan University, Wuhan, Hubei, China
e-mail: ssdu@whu.edu.cn

Supplementary Figures

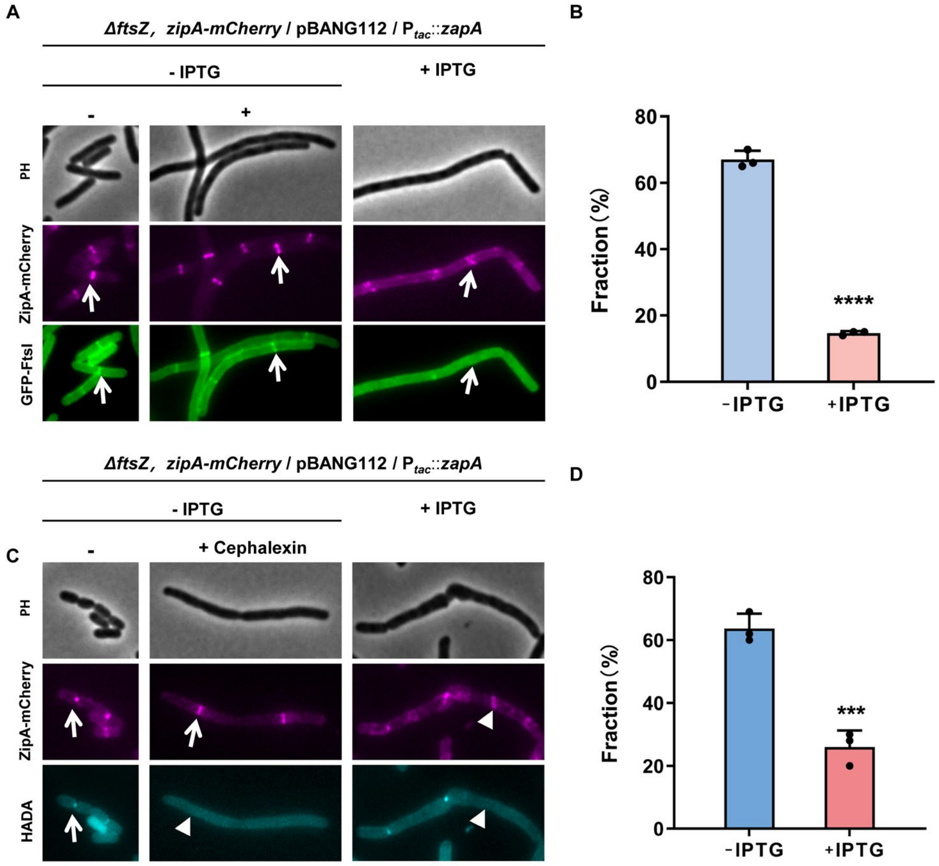

Fig. S1 ZapA overexpression blocks divisome assembly and septal peptidoglycan synthesis. (A-B) Representative images of ZipA-mCherry and GFP-FtsI localization (A) and quantitation of their co-localization (B) in the absence or presence of ZapA overexpression. Plasmid pCY129 (P_{tet}::*gfp-ftsI*) was transformed into strain CYa35 (W3110, *ftsZ⁰ zipA-mCherry /pACYC, ftsZ & pEXT22,* P_{tac}::*zapA*). Cells were grown with or without 500 µM IPTG for 1 h and samples visualized. Number of cells analyzed (-IPTG: n=674, +IPTG: n=171). (C-D) Representative images of ZipA-mCherry and HADA localization (C) and quantitation (D) of their co-localization in the absence or presence of ZapA overexpression. The cell cultures of CYa35 (W3110, *ftsZ⁰ zipA-mCherry /pACYC, ftsZ & pEXT22,* P_{tac}::*zapA*) were grown with or without 500 µM IPTG for 1 h, and incubated with HADA to label nascent PG. Number of cells analyzed (-IPTG: n=515, +IPTG: n=230). Asterisks denote a significant difference based on a P value of <0.0001 (****) in (B) and (D), and a P value of 0.0008 (***) in (J); ns, not significant, two-tailed Student's t test. Scale bars, 5 µm.

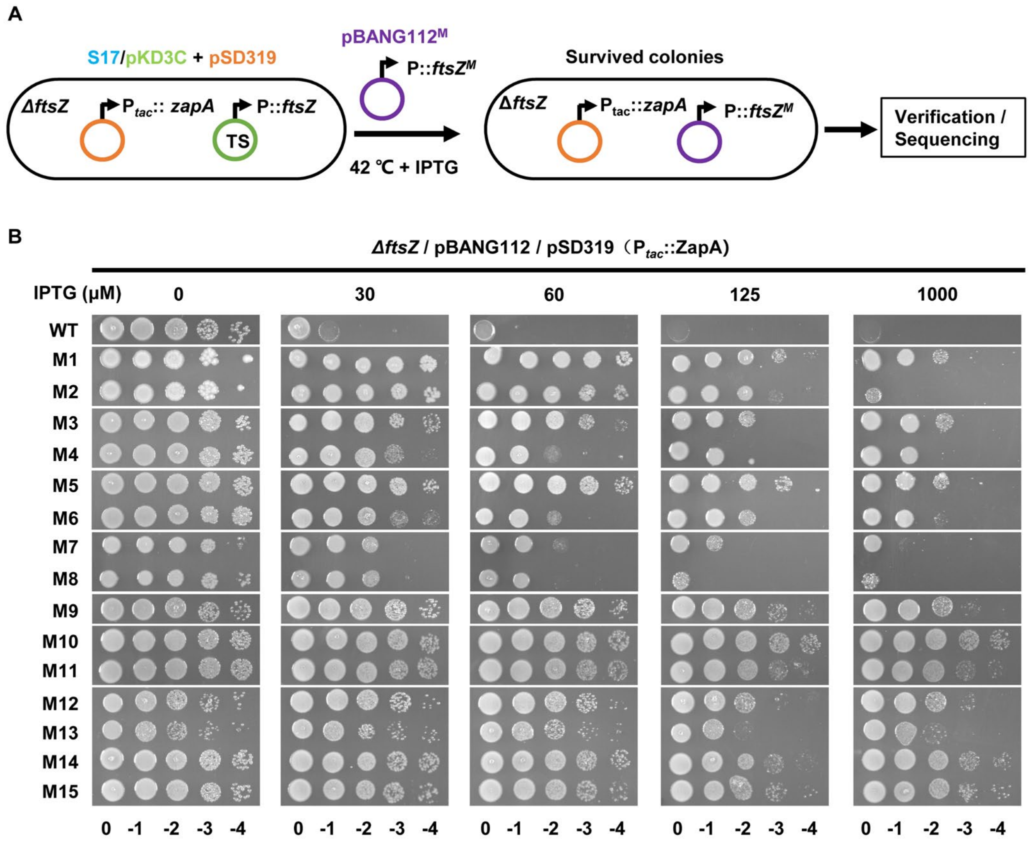

Fig. S2 Selection for FtsZ mutants resistant to ZapA overexpression toxicity. (A) A diagram showing the procedure for selection for FtsZ mutants. Plasmid pKD3C (in green) carrying *ftsZ* is temperature sensitive for replication, so it only complements the *ftsZ* deletion strain S17 at 30°C. The strain also harbors a plasmid expressing ZapA under an IPTG-inducible promoter (pSD319, in orange). The FtsZ mutant library (pBANG112^{M}, in purple) was introduced into the strain S17/pKD3C&pSD319 and transformants were selected in the presence of 30 μM IPTG at 42°C. Only transformants expressing functional FtsZ that provided resistance to ZapA overexpression would survive the selection. Surviving transformants were subjected to a spot test to confirm resistance to ZapA overexpression and *ftsZ* was sequenced to identify the mutations. (B) Verification of the FtsZ mutants resistant to ZapA overexpression before sequencing analysis. Transformants selected in (A) were subjected to a spot test on plates with increasing IPTG. Totally 15 mutants were isolated.

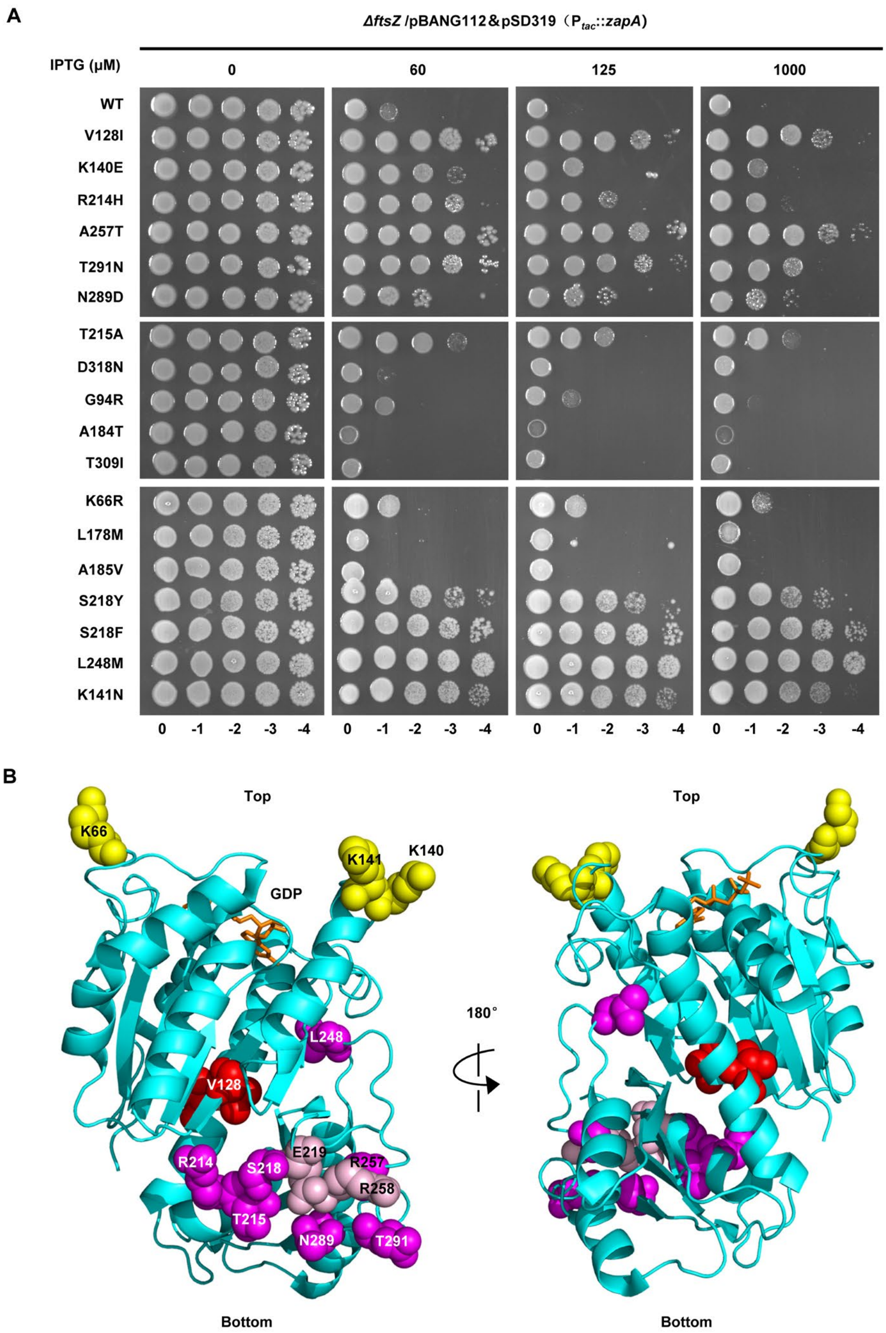

Fig. S3 Determination of the resistance of FtsZ single substitutions to ZapA overexpression toxicity. (A) Single substitutions identified in the 15 mutants isolated in Fig. S2B were re-introduced into pBANG112 by site-directed mutagenesis. The resultant pBANG112 derivatives carrying the single substitutions were transformed into S17/pKD3C&pSD319 and transformants selected at 42°C. Individual transformants

were subjected to a spot test on plates with increasing IPTG. (B) Locations of the mutated residues in the structure of *E. coli* FtsZ (PDB# 6UNX). Residues on the surface of FtsZ are colored yellow (top face) or magenta (bottom face), whereas residues buried inside FtsZ molecule are colored red. Mutations isolated by site-directed mutagenesis are colored pink. GTP is shown as stick in brown. Residues number are indicated.

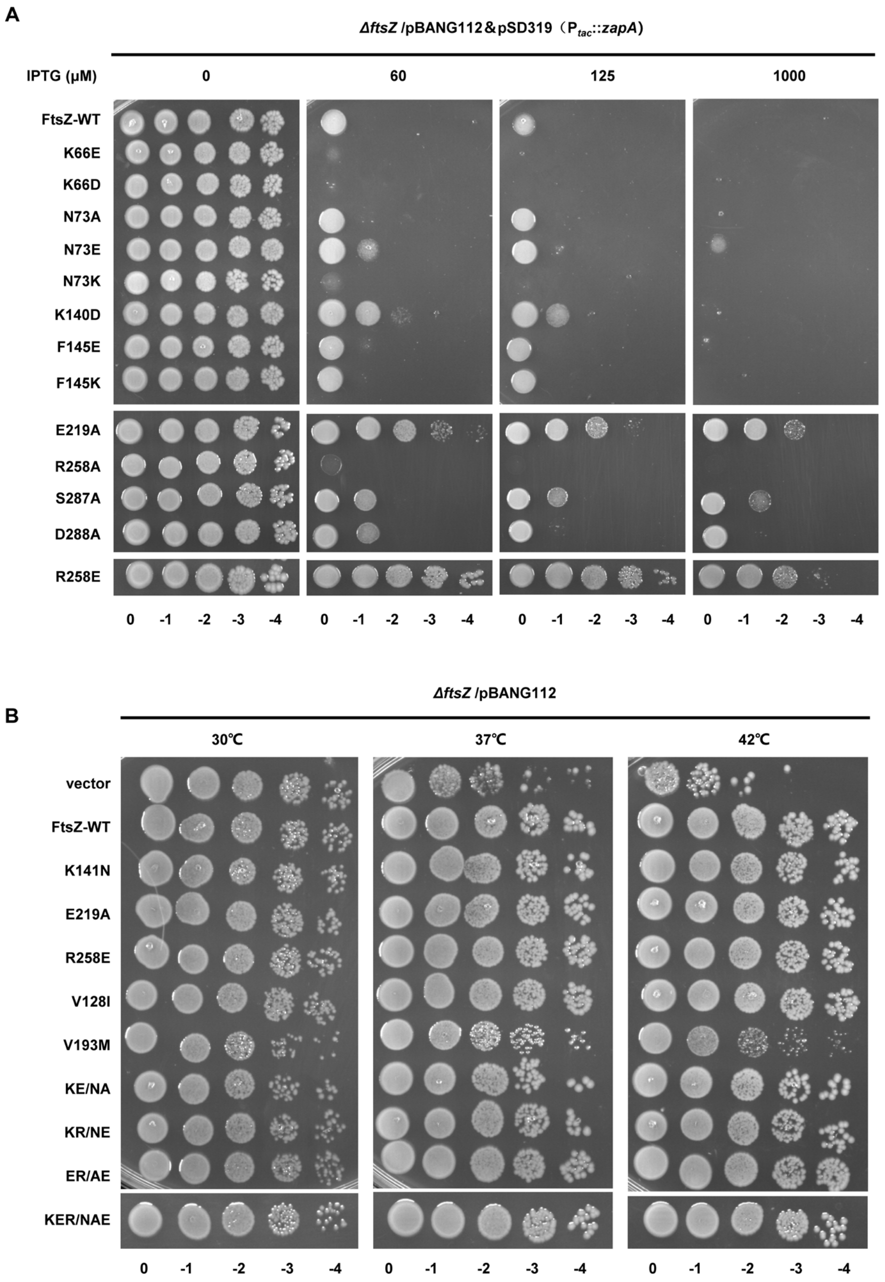

Fig. S4 Spot test of the resistance of FtsZ mutants to ZapA overexpression and their ability to complement FtsZ depletion strain. (A) Screening for additional FtsZ mutants providing resistance to ZapA overexpression. Residues adjacent to the mutated residues in Fig. S3 were replaced with the indicated amino acid in pBANG112. The variants were tested for resistance to ZapA overexpression as in Fig. S3A. Only E219A and R258E confer strong resistance to ZapA overexpression. (B) Complementation test of FtsZ mutants. Plasmid pKD3C carrying *ftsZ* is temperature sensitive for replication, so it only complements the FtsZ depletion strain S17 at 30°C. pBANG112 or its derivatives were transformed into S17/pKD3C at 30°C. Transformants were subjected to a spot test on plates at different temperatures.

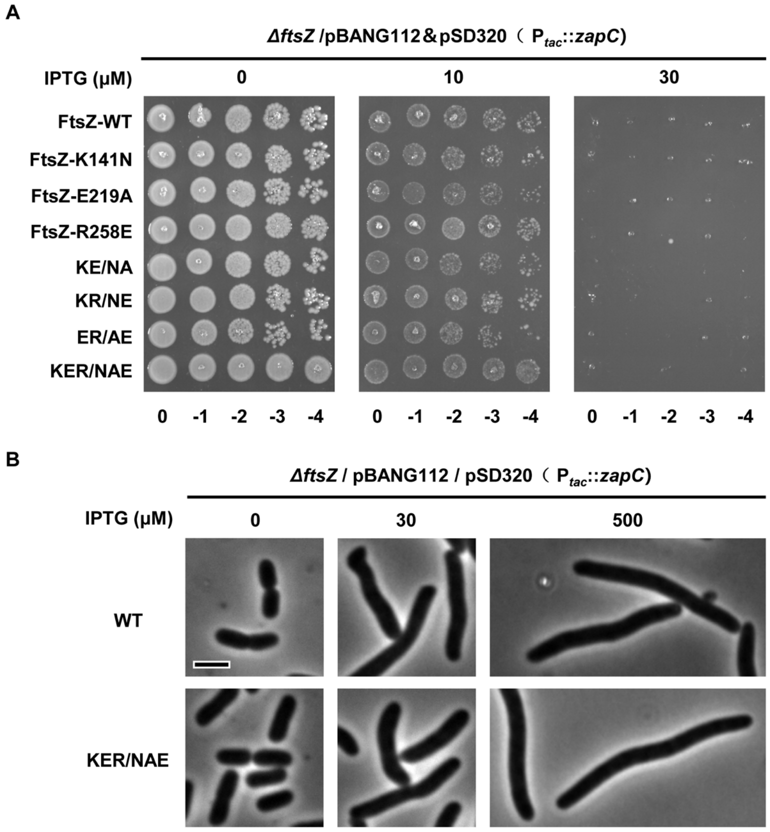

Fig. S5 FtsZ mutants do not provide resistance to overexpression of ZapC. (A) Spot test of the resistance of FtsZ mutants to ZapC overexpression. pBANG112 or its derivatives carrying the substitutions were transformed into S17/pKD3C&pSD320 (ZapC) and transformants selected at 42°C. Individual transformants were resuspended, serially diluted and spotted on plates with increasing concentration of IPTG at 37°C. (B) Representative image of the morphology of cells expressing FtsZ mutants and overexpressing ZapC in (A). Scale bar, 5 μm.

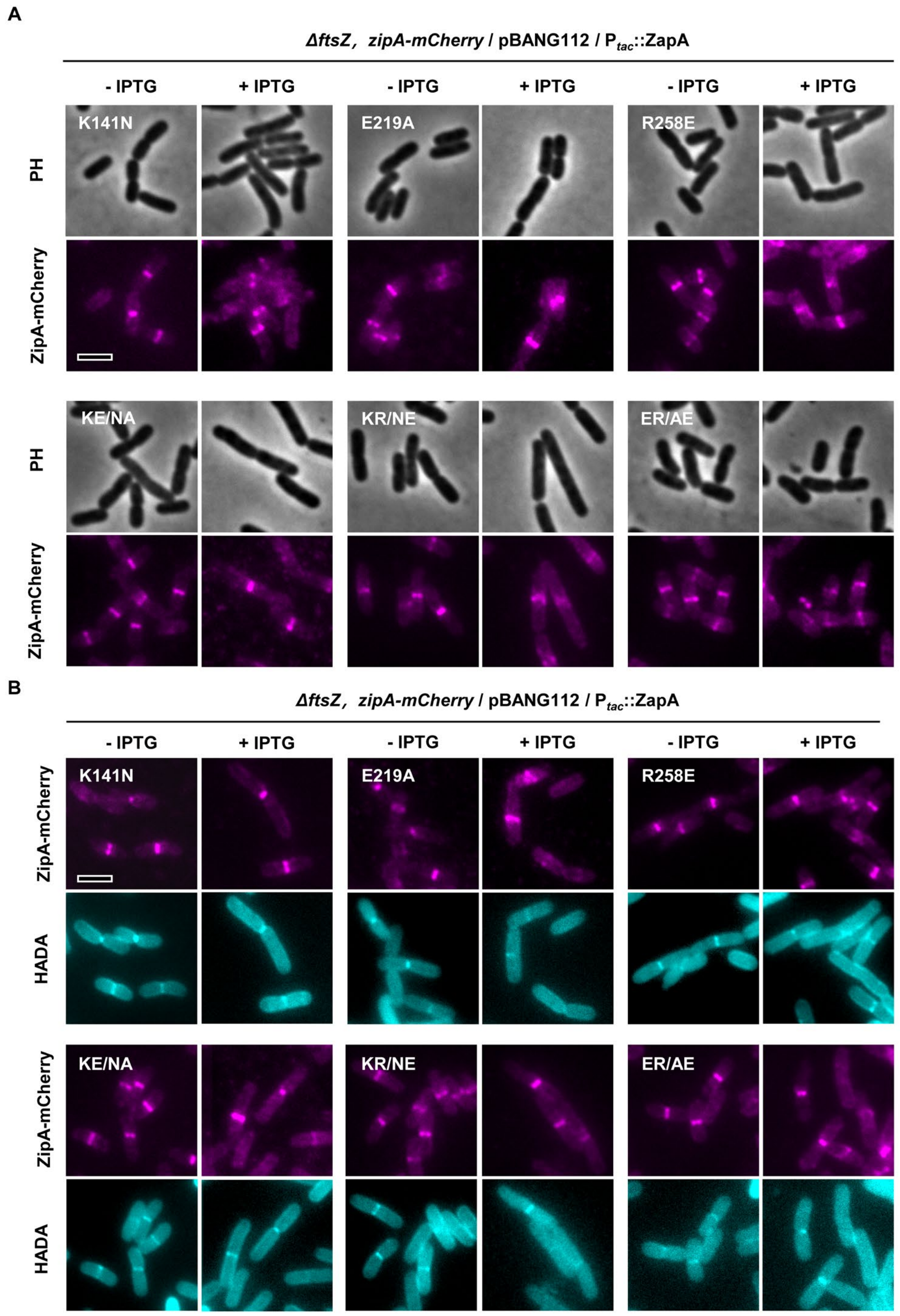

Fig. S6 Z rings formed by FtsZ mutants are resistant to ZapA overexpression. (A) Representative images of Z rings (ZipA-mCherry) in cells expressing FtsZ variants in the absence or presence of ZapA overexpression. Cells expressing FtsZ variants were grown in exponential phase, ZapA was induced with 500 μM IPTG, ZipA-mCherry was expressed from its native promoter. ZipA-mCherry was imaged by fluorescence microscopy. Corresponding to Fig. 3A. (B) Representative images of co-localization of ZipA-mCherry with HADA in cell expressing FtsZ variants in the absence or presence of ZapA overexpression. Strains were grown as in (A), nascent PG was labelled as described in Materials and Methods. Corresponding to Fig. 3B. Scale bars, 5 μm.

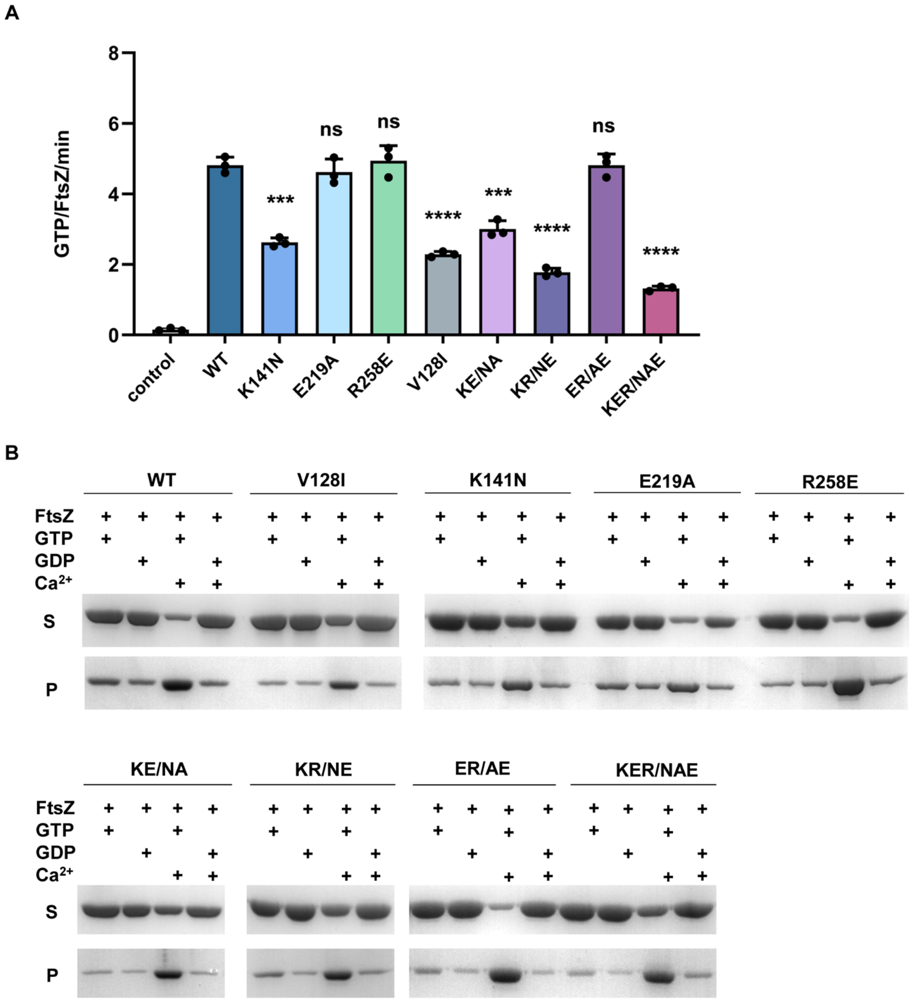

Fig. S7 FtsZ mutants retain the ability to polymerize and hydrolyze GTP. (A) GTPase activity of FtsZ mutants measured by the NADH coupled enzymatic assay. The reactions were carried out as described in Materials and Methods. FtsZ or the FtsZ mutants were added to a final concentration of 2.5 μM. The data was collected and then plotted using the Prism software, and the reaction rates were calculated using the Beer-Lambert law (A= ε×c×l). Asterisks denote a significant difference based on a P value of <0.0001 (****), 0.0001< P <0.008 (***), ns: not significant, two-tailed Student's t test. (B) Sedimentation assay to access the polymerization ability of FtsZ mutants. FtsZ or its variant (5 μM) were added into polymerization buffer (50 mM HEPES pH 6.8, 10 mM $MgCl_2$, 200 mM KCl) in the presence of and GTP (2.5 mM) and 10 mM Ca^{2+} in a 50 μL reaction volume. The samples were incubated at room temperature for 5 min before centrifuged and the pellets and supernatants were analyzed by SDS-PAGE. KE/NA: K141N, E219A; KR/NE: K141N, R258E; ER/AE: E219A, R258E. Scale bar, 0.2 μm.

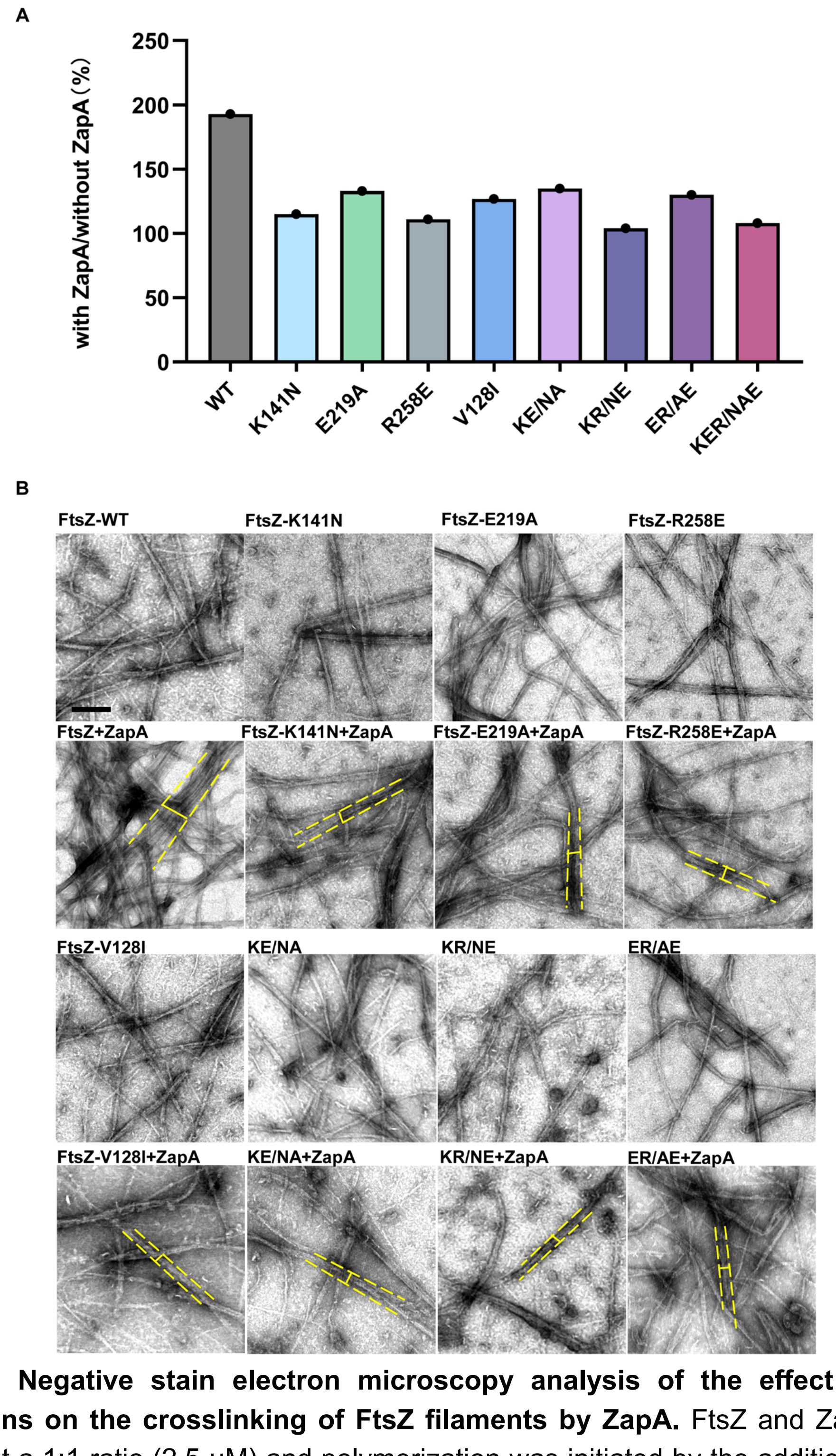

Fig. S8 Negative stain electron microscopy analysis of the effect of FtsZ mutations on the crosslinking of FtsZ filaments by ZapA. FtsZ and ZapA were added at a 1:1 ratio (2.5 μM) and polymerization was initiated by the addition of GTP (1 mM). After incubation at room temperature for 5 min, samples were dropped onto discharged grids and stained with 1% uranyl acetate. FtsZ filaments were examined by negative stain electron microscopy. Yellow lines show the width of FtsZ bundles. KE/NA: K141N, E219A; KR/NE: K141N, R258E; ER/AE: E219A, R258E; Scale bar, 0.2 μm.

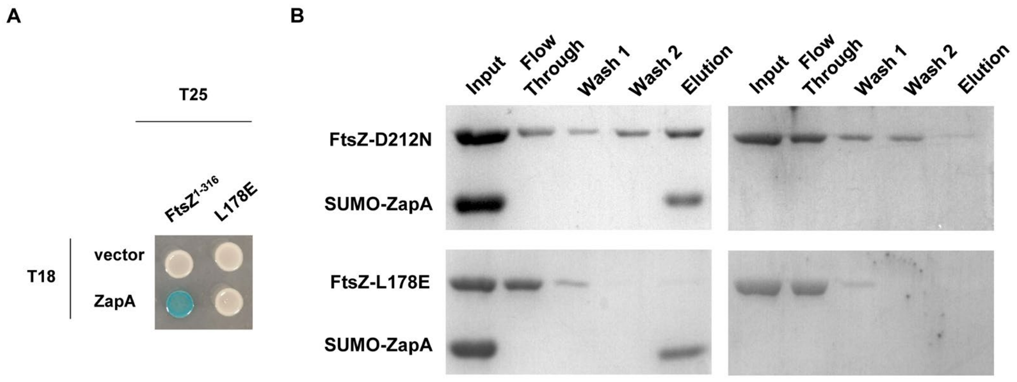

Fig. S9 ZapA prefers to bind to polymerized FtsZ instead of monomeric FtsZ. (A) BTH assay to access the impact of the mutation L178E on the interaction between $FtsZ^{1\text{-}316}$ and ZapA. Pairs of plasmids expressing the indicated fusions to the T18 and T25 domains of adenine cyclase were transformed into strain BTH101. A single transformant was resuspended in 1 mL of LB solution and spotted on LB plates containing IPTG-Xgal. Plates were incubated at 30°C for 12 h prior imaging. Blue indicates a positive interaction. (B) Pull-down assay to test the interaction between ZapA and stable FtsZ filaments ($FtsZ^{D212N}$) or monomeric $FtsZ^{L178E}$. Assays were performed as Fig. 5C.

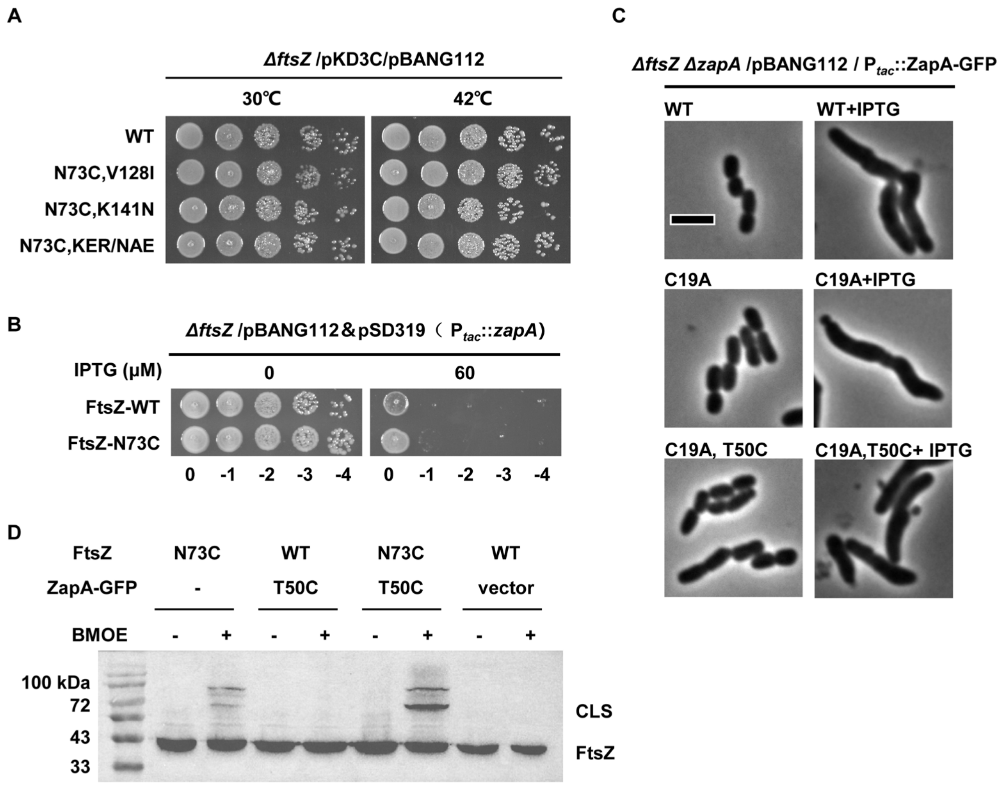

Fig. S10 *In vivo* BMOE crosslinking assay to access the interaction between FtsZ and ZapA. (A) Spot test to check the effect of the N73C mutation on the ability of FtsZ to complement. pBANG112 or its derivatives expressing the indicated FtsZ variants were transformed into an FtsZ depletion strain. Transformants were selected at 30°C and then subjected to a spot test at 30°C and 42°C. (B) Spot test to check the effect of N73C mutation on the sensitivity of FtsZ to ZapA overexpression. pBANG112 or its derivative expressing FtsZ^{N73C} were transformed into an FtsZ depletion strain harboring a plasmid expressing ZapA. Transformants were selected at 42°C and then subjected to a spot test on plates with or without IPTG at 37°C. (C) Introduction of the T50C mutation into ZapA does not reduce its overexpression toxicity. pCY170 (P$_{tac}$::*zapA-gfp*) or its derivatives were transformed into CYm138 (*W3110, ftsZ::cam /pACYC, ftsZ*, *zapA<>frt*) . The cells were grown with or without 500 μM IPTG for 1 h and the cultures were imaged by microscopy. Scale bar, 5 μm. (D) *In vivo* BMOE crosslinking assay to test the interaction between FtsZ^{N73C} and ZapA$^{(C19A)-T50C}$. Strains expressing the indicated form of FtsZ and ZapA-GFP were grown in LB in exponential phase, treated with BMOE or DMF as described in Materials and Methods. Samples were treated with β-mercaptoethanol and then cells were harvested by centrifugation and prepared for SDS-PAGE and western blot. Samples with both FtsZ and ZapA-GFP cysteine mutant pairs exhibited a significant band at 72-100 kDa corresponding to a crosslinked FtsZ-ZapA-GFP polypeptide. CLS: crosslinked species; NCLS: non-specific crosslinked species.

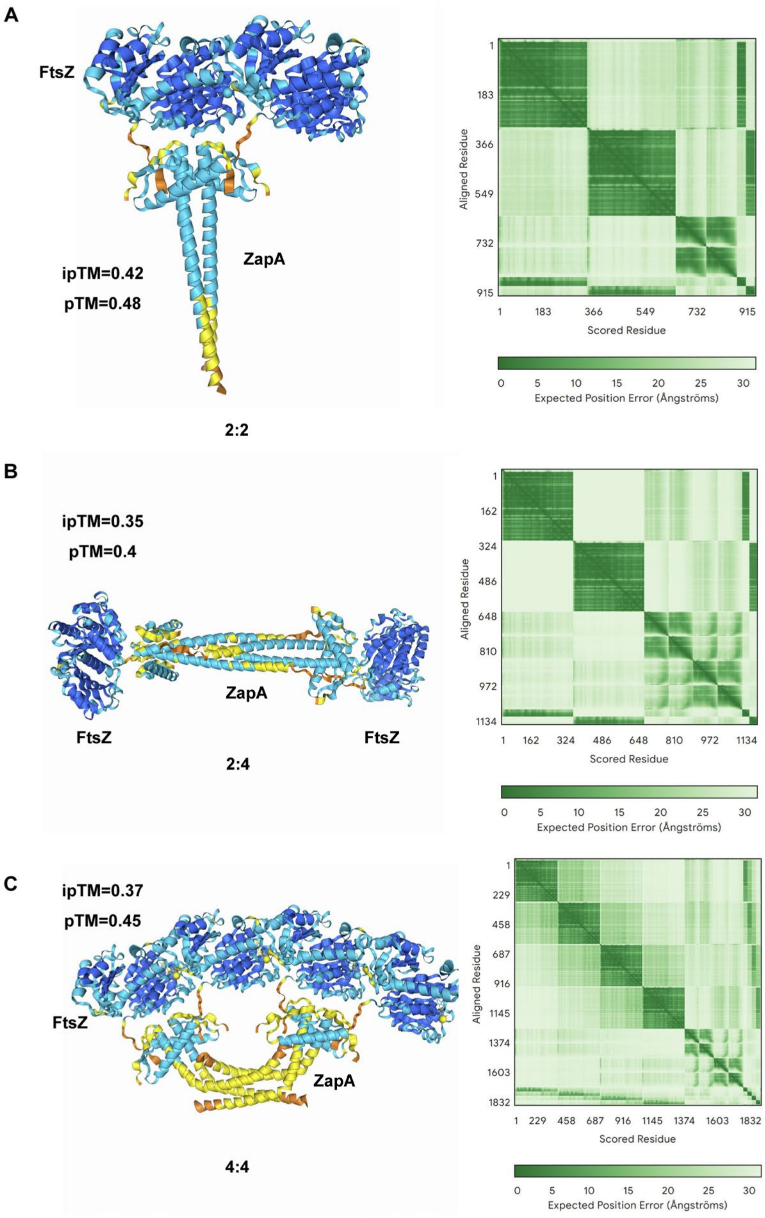

Fig. S11 Structural models of FtsZ-ZapA complex generated by AlphaFold 3. (A-C) AlphaFold 3 model of the FtsZ-ZapA complex at ratios of 2:2, 2:4; 4:4 between FtsZ and ZapA. Regardless of the ratio, the N-terminal motif of FtsZ binds to a groove in the dimer head of ZapA tetramer. ipTM, pTM values and PAE for each model were indicated and shown.

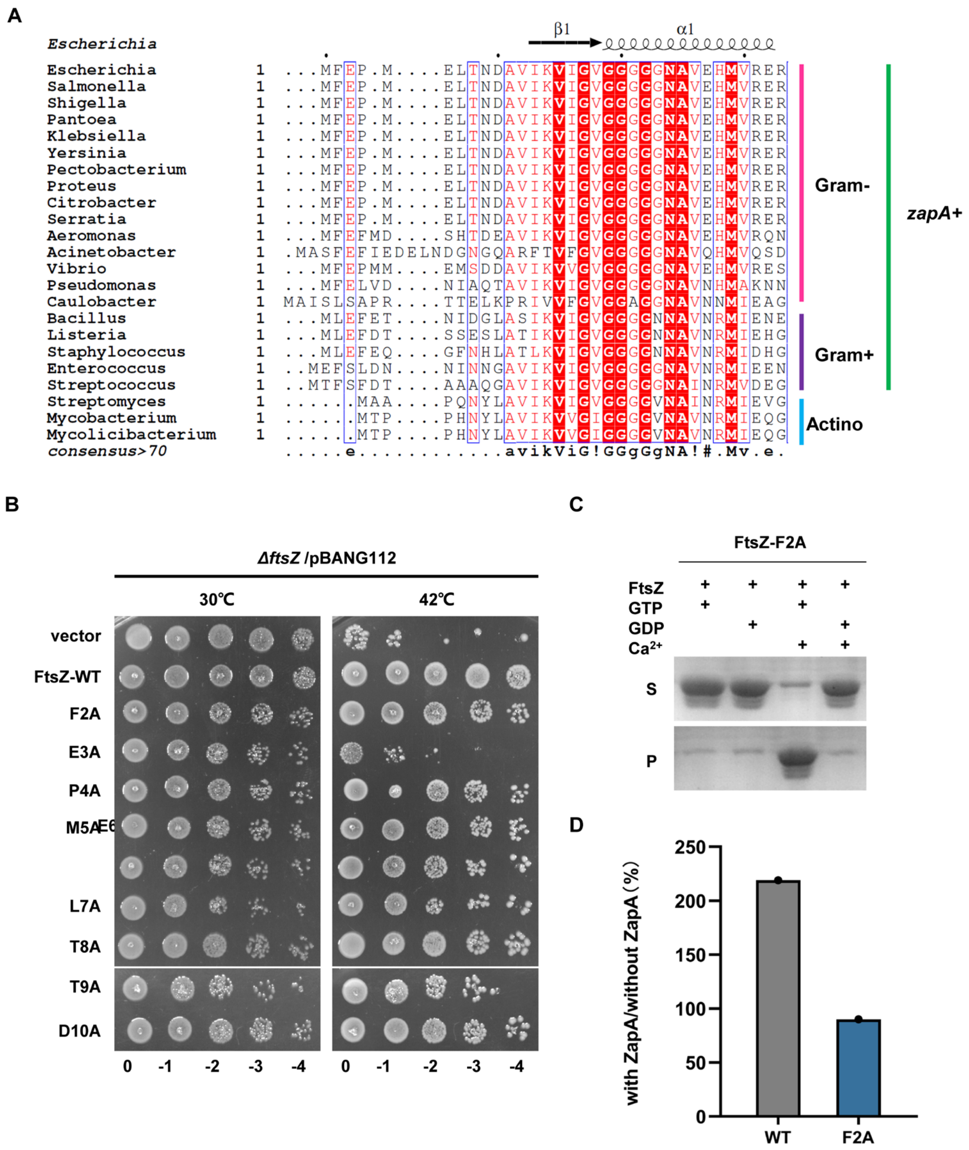

Fig. S12 Alignment of the N-terminal sequence of FtsZ proteins and characterization of FtsZ mutants. (A) Alignment of the N-terminal amino acid sequences of FtsZ proteins from diverse bacteria. In both Gram+ and Gram- bacteria harboring *zapA*, a conserved motif is present at the N-terminus, featured by a large hydrophobic amino acid (F/L) and a glutamate at the second and third position (*E. coli* numbering), respectively, whereas in actinobacteria which do not encode for ZapA these residues are missing. (B) Complementation test of FtsZ N-terminal mutants. Plasmid pKD3C carrying *ftsZ* is temperature sensitive for replication, so it only complements the *ftsZ* defective strain S17 at 30°C. pBANG112 or its derivatives were transformed into S17/pKD3C at 30°C. Transformants were subjected to a spot test on plates at 30 °C or 42°C overnight. (C) Sedimentation assay to test the polymerization of FtsZ^{F2A}. FtsZ or FtsZ^{F2A} (5 μM) were mixed in polymerization buffer (50 mM HEPES pH 6.8, 10 mM $MgCl_2$, 200 mM KCl) in the presence of GTP (2.5 mM) and Ca^{2+} in a 50 μL reaction volume. The samples were incubated at room temperature for 5 min

before being centrifuged and the pellets and supernatants were analyzed by SDS-PAGE. (D) Quantification of the effect on the F2A mutation on the sedimentation efficiency of FtsZ by ZapA. Corresponding to Fig. 8C.

Table S1. FtsZ mutants isolated by genetic selection.

Mutant number	*ftsZ* alleles	Complementation	Resistance to ZapA (IPTG: μM)
M1	V128I	YES	1000
M2	K140E	YES	125
M3	G191C,A257T	YES	1000
M4	R214H	YES	125
M5	T291N	YES	1000
M6	T215A,D318N	YES	125
M7	N289D	YES	125
M8	G94R,A184T,T309I	YES	125
M9	K66R,A185V	YES	1000
M10	S218Y	YES	1000
M11	L248M	YES	1000
M12	A184T,V193M	YES	1000
M13	V193M	YES	1000
M14	K141N	YES	1000
M15	L178M,S218F	YES	1000

Table S2. FtsZ single substitutions with significant resistance to ZapA overexpression.

ftsZ alleles	Complementation	Resistance to ZapA (IPTG: μM)	Resistance to ZapC	Source
WT	YES	NO	NO	Collection
V128I	YES	1000	NO	This study
K141N	YES	1000	NO	This study
V193M	YES	1000	NO	This study
S218F/Y	YES	1000	NO	This study
E219A	YES	1000	NO	This study
L248M	YES	1000	NO	This study
A257T	YES	1000	NO	This study
R258E	YES	1000	NO	This study
T291N	YES	1000	NO	This study
K66R	YES	125	NO	This study
K140E/D	YES	125	NO	This study
R214H	YES	125	NO	This study
T215A	YES	125	NO	This study
N289D	YES	125	NO	This study

Table S3. List of strains used in this study.

Strain	Description	Source/Reference
BL21	*F – ompT hsdSB (rB- mB-) gal dcm* (DE3)	Lab collection
BTH101	*F- cya-99, araD139 galE15 galK16 rpsL1 (StrR) hsdR2 mcrA1 mcrB1*	Lab collection
CYa35	W3110, *ftsZ*^{0}, *zipA-mcherry-spc* / pACYC, *ftsZ* & pEXT22, *P*$_{tac}$*::zapA*	This study
CYb1	W3110,*ftsZ*^{0} *,zapA-gfp-cat, zapB::kan* /pACYC, *ftsZ*	This study
CYm141	W3110, *ftsZ::cam, zapA<>frt* /pACYC, *ftsZ*^{N73C}	This study
CYm142	W3110/pZH509,*ftsZ-linker5-mNG*	This study
CYm143	W3110/pZH509,*ftsZ-linker5-mNG*&pEXT22,*P*$_{tac}$*::zapA*	This study
EC436	MC4100, *(λattL-lom)::bla lacIqP*$_{207}$*-gfp-ftsI*	Lab collection
JS238	MC1061, *malPp::lacIq srlC::Tn10 recA1*	Lab collection
S17	W3110, *ftsZ*^{0}	Lab collection
W3110	*F- λ- rph-1 INV(rrnD, rrnE)*	Lab collection

Table S4. List of plasmids used in this study.

Plasmid	Description	Source/Reference
pBANG112	pACYC, *ftsZ*, Amp^r	(1)
pBC1	pACYC, *ftsZ*^{N73C}, Amp^r	This study
pCY52	pE-SUMO,P_{T7}*::his-SUMO-ZapA*, Amp^r	This study
pCY54	pE-SUMO,P_{T7}*::his-SUMO-ftsZ*^{L178E}, Amp^r	This study
pCY55	pE-SUMO,P_{T7}*::his-SUMO-ftsZ*^{D212N}, Amp^r	This study
pCY83	pKNT25, P_{lac}*::ftsZ*$^{1\text{-}316}$*-T25*, Kan^r	This study
pCY86	pKNT25, P_{lac}*::ftsZ*$^{317\text{-}383}$*-T25*, Kan^r	This study
pCY170	pEXT22, P_{tac}*::zapA-gfp*, Kan^r	This study
pCY173	pEXT22, P_{tac}*::zapA*^{C19A,T50C}*-gfp*, Kan^r	This study
pCY205	pZH509*, gfp-ftsI,cat,* Amp^r	This study
pDSW210	pDSW206-MCS-*gfp*, Amp^r	(2)
pE-SUMO-Amp	pUC, P_{T7}*::6XHis-SUMO-MCS*, Amp^r	Lab collection
pE-SUMO-FtsZ	pE-SUMO,P_{T7}*::his-SUMO-ftsZ*, Amp^r	Lab collection
pKD3C	pSC101, *repA*^{ts} *ftsZ* Cam^r	(3)
pKNT25	P_{lac}*::T25*, Kan^r	Lab collection
pSD319	pEXT22, P_{tac}*::zapA*, Kan^r	Lab collection
pSD320	pEXT22, P_{tac}*::zapC*, Kan^r	Lab collection
pSY3	P15A, bla terR $P_{LtetO\text{-}1}$::*ftsZ-mneongreen*	This study
pT18A	pUT18, P_{lac}*::zapA-T18*, Amp^r	This study
pT18-zip	pBluescript II KS, T18-leucine zipper	(4)
pT25-zip	pACYC184, *lac UV5T25-leucine zipper*	(4)
pUT18C	*Plac::T18*, Amp^r	Lab collection

pXY027	ColE1, *cat lacI*^{Q} P_{T5lac}::*ftsZ-gfp*	(5)
pYD208	P15A, *bla terR* $P_{LtetO-1}$::*ftsW-mneongreen*	Lab collection
pZH509-gfp	P15A, *bla terR* $P_{LtetO-1}$*::gfp*	(6)
pZT25	pKNT25, P_{lac}*::ftsZ-T25*, Kan^{r}	Lab collection

Table S5. Primers used in this study

Primer name	Sequence
FtsZ-N73C-F	CTGGGCGCTGGCGCTTGTCCAGAAGTTGGCCGC
FtsZ-N73C-R	GCGGCCAACTTCTGGACAAGCGCCAGCGCCCAG
T18-ZapA-F	CGCGGATCCATCTGCACAACCCGTCGATAT
T18-ZapA-R	CGGGGTACCTCATTCAAAGTTTTGGTTAG
BsaI-ZapA-F	CGGGTCTCTAGGTTCTGCACAACCCGTCGATAT
XbaI-ZapA-R	CGCTCTAGATTATTCAAAGTTTTGGTTAGTTTTTTCGGT
FtsZ-L178E-F	GGCCGCGGTATCTCCGAGCTGGATGCGTTTGGC
FtsZ-L178E-R	GCCAAACGCATCCAGCTCGGAGATACCGCGGCC
FtsZ-D212N-F	GAACGTGGACTTTGCAAACGTACGCACCGTAATG
FtsZ-D212N-R	CATTACGGTGCGTACGTTTGCAAAGTCCACGTTC
SalI-ZapA-F	ACGCGTCGACGACGTCTCTGCACAACCCGTCGATAT
ZapA-HindIII-R	CCCAAGCTTAAAGTGTCATTCAAAGTTTTGGTTAGTTTT
pZT25-F	CCCAAGCTTGTAGGCGACAGGCACAAATCGGAG
316-XbaI-R	CGTCTAGAGCGCCGATACCTGTCGCAAC
HindIII-317-F	GCAAGCTTATGGGCATGGACAAACGTCCT

383-XbaI-R	CGTCTAGAGCATCAGCTTGCTTACGCAG
XbaI-GFP-F	CGTCTAGAATGAGTAAAGGAGAAGAACTTTTCAC
FtsI-HindIII-R	CGAAGCTTGTCGCGCAAATTACGATCTG
EcoRI-ZapA-F	CGGAATTCTAATCAATCAGCAGGAAGGT
ZapA-GFP-R	CTTCTCCTTTACTCATCGAAAAGTGTTCAAAGTTTTGG
ZapA-GFP-F	CTTTGAACACTTTTCGATGAGTAAAGGAGAAGAACTTTTC
GFP-HindIII-R	CGAAGCTTTTATTTGTATAGTTCATCCATGCCATG
509-GFPU-F	CATTAAAGAGGAGAAAGGTACCATGAGTAAAGGAGAAGAACT
FtsI-C-R	CCAGTGATTTTTTTCTCCATTCTAGATTACGATCTGCCACCT
FtsI-C-F	AGGTGGCAGATCGTAATCTAGAATGGAGAAAAAAATCACTGG
C-509U-R	TAGCACGCGTACCATGGGATCCTTACGCCCCGCCCTG
ZapA-C19A-F	GTTCACTGCGTGTGAACGCGCCGCCTGACCAAAGG
ZapA-C19A-R	CCTTTGGTCAGGCGGCGCGTTCACACGCAGTGAAC
ZapA-T50C-F	CACTAGAGTCACAAATTGTGAACAGTTGGTCTTC
ZapA-T50C-R	GAAGACCAACTGTTCACAATTTGTGACTCTAGTG
FtsZ-F2A-F	CAAATCGGAGAGAAACTATGGCGGAACCAATGGAACTTACC
FtsZ-F2A-R	GGTAAGTTCCATTGGTTCCGCCATAGTTTCTCTCCGATTTG
FtsZ-V128I-F	GATTTGGGTATCCTGACCATTGCTGTCGTCACTAAGC
FtsZ-V128I-R	GCTTAGTGACGACAGCAATGGTCAGGATACCCAAATC
FtsZ-K141N-F	CAACTTTGAAGGCAAGAATCGTATGGCATTCGCG
FtsZ-K141N-R	CGCGAATGCCATACGATTCTTGCCTTCAAAGTTG
FtsZ-E219A-F	CGCACCGTAATGTCTGCGATGGGCTACGCAATG

FtsZ-E219A-R	CATTGCGTAGCCCATCGCAGACATTACGGTGCG
FtsZ-R258E-F	GACCTGTCTGGCGCGGAAGGCGTGCTGGTTAACATC
FtsZ-R258E-R	GATGTTAACCAGCACGCCTTCCGCGCCAGACAGGTC
oSY1	GTGAGCAAAGGCGAAGAAGATAAC
oSY2	GCGGTACCTTTCTCCTCTTTAATG
oSY3	GAGGAGAAAGGTACCGCATGTTTGAACCAATGGAACTTAC
oSY4	TTCGCCTTTGCTCACCCTTCGAATGTGTATCTTGCCTATTCT GCCATCAGCTTGCTTACGC

Construction of strains and plasmids

Strain CYa35 (*ftsZ⁰/pACYC, ftsZ & pEXT22,* P_{tac}*::zapA, zipA-mCherry-spc*) was constructed in two steps. Frist, plasmid pBANG112 and plasmid pSD319 were introduced into the FtsZ depletion strain S17/pKD3C (*ftsZ⁰ /pSC101ᵗˢ, ftsZ*). Colonies were selected at 42°C on plates containing ampicillin, kanamycin and glucose. The transformants were then streaked on LB plates with ampicillin and kanamycin at 42°C, followed by testing for sensitivity to chloramphenicol to verify plasmid pKD3C had been cured. The resultant strain was named CYm1 (*ftsZ⁰ /pACYC, ftsZ & pEXT22,* P_{tac}*::zapA*). The *zipA-mCherry-spc* cassette was then transduced into strain CYm1 using P1 grown on CYa13 (*zipA-mCherry-spc*). The purified transductant was checked for ZipA localization and named CYa35. Strains carrying other *ftsZ* alleles and *zipA-mCherry* were constructed similarly using derivatives of pBANG112.

Strain CYb1 (*ftsZ⁰/pACYC, ftsZ*, *zapA-gfp-cat, zapB::kan*) was constructed in several steps. First strain S17/pBANG112 (*ftsZ⁰ /pACYC, ftsZ*) was constructed similarly to the CYm1. The resultant strain was then transduced with P1 from HC261 (*zapA-gfp-cat*) and transductants were selected on LB plates containing chloramphenicol. The purified transductant was named CYa1 (*ftsZ⁰, zapA-gfp-cat /pACYC, ftsZ*). Finally, the *zapB::kan* allele from SD210 (W3110, *zapB::kan*) was transduced into CYa1 by selecting for kanamycin resistance on LB plates. The resultant transductant was sequenced and named CYb1 (*ftsZ⁰,zapA-gfp-cat, zapB::kan /pACYC, ftsZ*). Strains carrying other *ftsZ* alleles and expressing *zapA-gfp* were constructed similarly using derivatives of pBANG112.

Strains for in vivo crosslinking experiments were constructed in two steps, exemplified by CYm141 (*ftsZ::cat, zapA<>frt /pACYC,* $ftsZ^{N73C}$). First, plasmid pBC1 (*pACYC,* $ftsZ^{N73C}$) was introduced into the strain SH19 (W3310, *zapA<>frt*). Colonies were selected on plates containing ampicillin. Second, the *ftsZ::cat* allele from CYm102 was transduced into SH19/pBC1 by selecting for chloramphenicol resistance on LB plates. The resultant transductant was sequenced and named CYm141. Strains expressing other *ftsZ* alleles were constructed similarly using derivatives of pBANG112.

Strains CYm142 (W3110 */pZH509-FtsZ-linker5-mNG*) was constructed by introducing pSY3 (*pZH509-FtsZ-linker5-mNG*) into the strain W3110. Colonies were selected on plates containing ampicillin. The purified transformant was named CYm142. Plasmid pSD319 (*pEXT22,* P_{tac}*::zapA)* was introduced into the strain CYm142. Colonies were selected on plates containing kanamycin. The resultant transformant was named CYm143.

Plasmid pBC1 was constructed by site-directed mutagenesis using plasmid pBANG112 as the template and primer pairs FtsZ-N73C-F/R. Based on complementation tests, the cysteine substitution did not affect the function of FtsZ.

Plasmid pT18A was constructed by ligation of a BamHI/KpnI digested DNA fragment carrying the *zapA* coding sequence into pUT18C digested with the same enzymes. The DNA fragment was amplified from W3110 using primers T18-ZapA-F and T18-ZapA-R.

Plasmid pCY52 was constructed by ligation of a BsaI/XbaI digested DNA fragment carrying the *zapA* coding sequence into pE-SUMO-Amp digested with BsaI. The DNA fragment was amplified from W3110 using primers BsaI-ZapA-F and XbaI-ZapA-R.

Plasmid pCY54 and pCY55 were constructed by site-directed mutagenesis using plasmid pE-SUMO-FtsZ as the template and primer pairs FtsZ-L178E-F/R and ftsZ-D212N-F/R. These primers were listed in Table S5.

Plasmid pCY83 was constructed by ligation of a HindIII/XbaI digested DNA fragment carrying the $ftsZ^{1\text{-}316}$ coding sequence into pKNT25 digested with the same enzymes. The DNA fragment was amplified from pZT25 using primers pZT25-F and 316-XbaI-R.

Plasmid pCY86 was constructed by ligation of a HindIII/XbaI digested DNA fragment carrying the $ftsZ^{317\text{-}383}$ coding sequence into pKNT25 digested with the same enzymes. The DNA fragment was amplified from pZT25 using primers HindIII-317-F and 383-XbaI-R.

Plasmid pCY170 was constructed by replacing the *zapA* coding sequence of pSD319

with the coding sequence of *zapA-gfp*. To do this, the coding sequence of *zapA* was amplified from W3110 using primer EcoRI-ZapA-F and ZapA-GFP-R. The *gfp* fragment was amplified from pDSW210 using primer ZapA-GFP-F and GFP-HindIII-R. Overlap PCR used the purified PCR products as the template and primer pairs EcoRI-ZapA-F and GFP-HindIII-R. The resulting PCR fragment contains a fused *zapA-gfp* coding sequence, and was digested with EcoRI /HindIII and ligated into pSD319 cut with same enzymes.

Plasmid pCY173 was constructed by site-directed mutagenesis using plasmid pCY170 as the template and primer pairs ZapA-C19A-F/R and ZapA-T50C-F/R.

For plasmid pCY205 the coding sequence of *gfp-ftsI* was amplified from EC436 using primer 509-GFPU-F and FtsI-C-R. The *cat* fragment was amplified from pBAD33 using primer FtsI-C-F and C-509U-R. Overlap PCR was then used and the purified PCR products as the template with primer pairs 509-GFPU-F and C-509U-R. The resulting PCR fragment contains *gfp-ftsI* and *cat* coding sequence, and was ligated into BamHI/KpnI digested pSY3 by U-clone.

Plasmid pSY3 was constructed by seamless cloning using ClonExpress II One Step Cloning Kit (Vazyme). To generate this plasmid, the *ftsW* gene and linker (GGGGSPAPAPGGGGS) were replaced by the *ftsZ* gene with a linker (GRIGKIHIRR) from the plasmid pYD028 (pZH509-*ftsW-mNG*)（lab stock, modified from pZH509-*gfp*, a gift from Dr. Zach Hensel) (69). The *ftsZ* gene was amplified from the plasmid pXY027 (pCH-*ftsZ-gfp*) (34) using primers oSY3 and oSY4, and the vector backbone was amplified from plasmid pYD028 using primers oSY1 and oSY2. These fragments were then joined to generate plasmid pSY3.

Derivatives of pBANG112, pBC1, pCY55, pCY83, pE-SUMO-FtsZ and pZT25 carrying different *ftsZ* mutations were created by site-directed mutagenesis using the primers listed in Table S5.

References:

1. Shen B & Lutkenhaus J (2009) The conserved C-terminal tail of FtsZ is required for the septal localization and division inhibitory activity of MinC(C)/MinD. *Mol Microbiol* 72(2):410-424.
2. Weiss DS, Chen JC, Ghigo JM, Boyd D, & Beckwith J (1999) Localization of FtsI (PBP3) to the septal ring requires its membrane anchor, the Z ring, FtsA, FtsQ, and FtsL. *J Bacteriol* 181(2):508-520.
3. Dai K & Lutkenhaus J (1991) ftsZ is an essential cell division gene in Escherichia coli. *J Bacteriol* 173(11):3500-3506.
4. Karimova G, Pidoux J, Ullmann A, & Ladant D (1998) A bacterial two-hybrid system based on a reconstituted signal transduction pathway. *Proc Natl Acad Sci U S A* 95(10):5752-5756.
5. Buss J*, et al.* (2015) A multi-layered protein network stabilizes the Escherichia coli FtsZ-ring and modulates constriction dynamics. *PLoS Genet* 11(4):e1005128.
6. Hensel Z (2017) A plasmid-based Escherichia coli gene expression system with cell-to-cell variation below the extrinsic noise limit. *PLoS One* 12(10):e0187259.
